# Digitally assessing social–emotional skills in early school years: initial validation of a screening instrument

**DOI:** 10.3389/fpsyg.2025.1529083

**Published:** 2025-02-06

**Authors:** Andrea Kogler, Barbara Gasteiger-Klicpera, Katharina Prinz, Lisa Paleczek

**Affiliations:** ^1^Inclusive Education Unit, Department of Education Research and Teacher Education, University of Graz, Graz, Austria; ^2^Research Center for Inclusive Education, Graz, Austria

**Keywords:** assessment, social–emotional skills, emotion regulation, primary school, digital instrument

## Abstract

**Introduction:**

Social–emotional skills are essential in everyday interaction and develop in early and middle childhood. However, there is no German instrument to help primary school students identify their strengths and weaknesses in different social–emotional skills that does not rely on written language. This paper introduces a new digital instrument, the GraSEF: Grazer Screening to assess Social–Emotional Skills, which was developed to measure (1) Behavior in Social Situations, (2) Prosocial Behavior, (3) Emotion Regulation Strategies, (4) Emotion Recognition and (5) Self-Perception of Emotions using different test formats (e.g., situational judgement test, self-assessment, performance tests). In the GraSEF, students work through an online survey tool, using audio instructions to guide them through the test.

**Methods:**

The present study analyses the responses of second graders (*M*_age_ = 8.23 years, *SD*_age_ = 0.48, 48% female). The intention was to gain initial insight into the instrument’s psychometric quality and user-friendliness.

**Results:**

In general, the instrument was found to have acceptable to good internal consistency, sufficient discriminatory power and item difficulty. However, one subtest (5: Self-Perception of Emotions), as well as three situations of the situational judgment test (1: Behavior in Social Situations), were excluded due to unsatisfactory fit and distribution. The validity check revealed low to moderate correlations between teacher rating and student scores. On average, students completed the screening in about 30 min and provided positive feedback regarding usability.

**Discussion:**

While the small sample size only provides preliminary insight into the instrument’s psychometric quality, the results suggest that the GraSEF can reliably measure various dimensions of social–emotional skills in second graders, even among those with low reading skills.

## 1 Introduction

Social–emotional competence is crucial for child development. It has a big impact on a child’s daily life experiences, especially in middle childhood. Promoting social–emotional skills in such a developmental period not only helps to prevent behavioral disorders. It is also likely to have a positive influence on child development, children’s prosocial behavior and well-being, and on their academic skills (Durlak et al., 2022; Greenberg et al., 2017; Taylor et al., 2017).

It is acknowledged that social–emotional competence is “a multidimensional construct that is critical to success in school and life for all children” (Domitrovich et al., 2017, p. 408). Nonetheless, there is a broad spectrum of definitions concerning social competence, emotional competence and social–emotional competence, with no clear consensus about the various facets involved (Wigelsworth et al., 2010; Gresham, 1986).

Social competence can be defined as effectiveness in social interactions from the perspective of the self and others (Gasteiger-Klicpera and Klicpera, 1999; Rose-Krasnor, 1997; von Salisch et al., 2022). A well-established framework for understanding social competence is the prism model of social competence (Rose-Krasnor, 1997). This model emphasizes context-dependence and exhibits three structural levels (skill level, index level, theoretical level). This topmost level is linked to the two lower levels: to achieve social effectiveness, we need to use the foundational skills appropriately and we need to be aware of the significance of different indicators of social competence (e.g., popularity or social status) (Rose-Krasnor, 1997).

Even though the model described above takes account of various facets of social competence, it does not explicitly consider emotional competence. One famous concept related to emotional competence is Emotional Intelligence (EI), introduced by Salovey and Mayer (1990). EI encompasses different abilities such as regulation of emotion, utilization of emotion, emotive regulation and evaluation and expression of emotions (Salovey and Mayer, 1990; Sergi et al., 2021). These abilities are critical for understanding the broader framework of social–emotional competence.

Social interactions entail many types of social skills (e.g., communication skills, prosocial behavior) as well as emotional skills (e.g., evaluating and expressing emotions, emotion regulation) and these skills are closely intertwined (Denham et al., 2002).

In order to combine both social and emotional competence, some frameworks focus on the acquisition of social–emotional skills (Soto et al., 2021), i.e., on social and emotional learning (SEL). The concept of SEL was developed by the Collaborative for Academic, Social, and Emotional Learning (CASEL) in the 1990s. The goal of SEL is to improve “children’s capacities to recognize and manage their emotions, appreciate the perspectives of others, establish prosocial goals and solve problems, and use a variety of interpersonal skills to effectively and ethically handle developmentally relevant tasks” (Payton et al., 2000, p. 179). Promoting SEL aims at enhancing five competences: (1) self-awareness, (2) self-management, (3) social-awareness, (4) relationship skills, and (5) responsible decision-making (Durlak et al., 2015).

**Figure 1:**
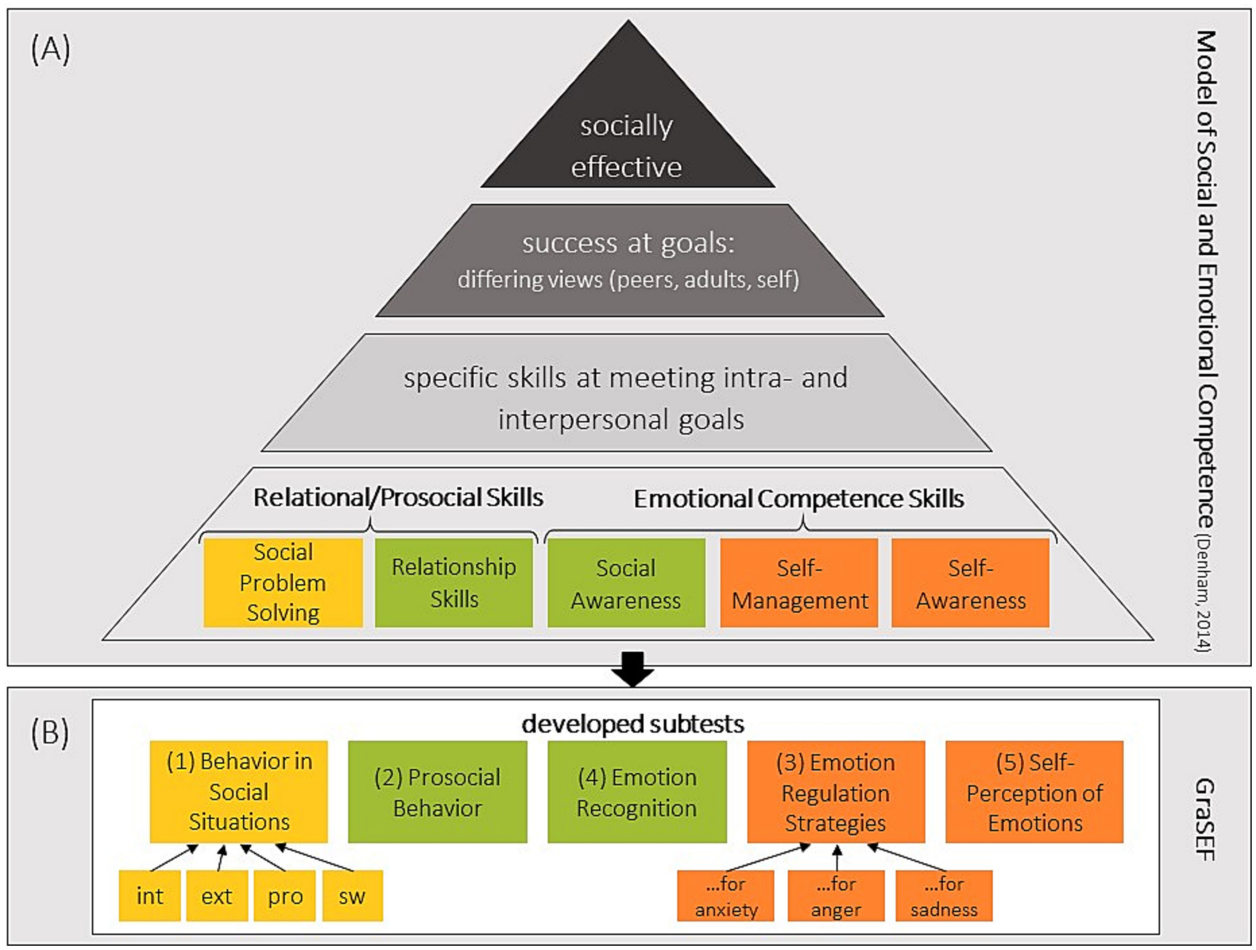
**(A)** Denham’s model of social and emotional competence (2014) based on Rose-Krasnor (1997) and Payton et al. (2000). **(B)** GraSEF’s subtests and subscales. int, internalizing behavior; ext, externalizing behavior; pro, problem-solving/assertive behavior; sw, social withdrawal.

The model (Denham et al., 2014), presented in Figure 1, combines the CASEL competences (Durlak et al., 2015; Payton et al., 2000) with an adapted form of the prism model of social competence (Rose-Krasnor, 1997). It differentiates between relational/prosocial skills (social problem solving, relationship skills) and emotional competence skills (self-awareness, self-management, social awareness). These skills are the basis of their triangle-shaped model. The next level of the model’s four levels refers to the specific skills needed in meeting intra- and interpersonal goals, which is the basis for goal success. The topmost level represents social effectiveness.

Many studies highlighted the role of social–emotional competence in child development. One meta-analysis for example, analyzed 82 SEL interventions from 1981 to 2014 (Taylor et al., 2017). The participants involved were kindergarten to high school students. They found that implementing programs that promote social–emotional skills could prevent the development of problems in child development (e.g., emotional distress, behavior problems, …) as well as improving positive attitudes, prosocial behavior and academic skills even in follow-up tests (Taylor et al., 2017). Another study measured social–emotional and behavioral skills and traits in adolescents from 15 to 20 years (*n* = 975). They found, that social–emotional skills as well as traits predict academic outcomes and highlighted the significance of skills and traits in predicting and understanding academic achievement (Soto et al., 2023).

### 1.1 Measuring social–emotional skills

In both research and educational practice, assessing the different aspects of SEL requires accurate instruments for measuring social–emotional skills. These instruments need to identify children with low competence, i.e., those who are at risk of developing behavioral problems. Furthermore, such instruments can be used to evaluate SEL-intervention efficiency (McKown, 2017). Hence, the development and improvement of such measurement instruments may be regarded as essential, particularly with respect to children in their early school years (Abrahams et al., 2019; Halle and Darling-Churchill, 2016).

Several reviews have critically evaluated the SEL-instruments available for preschool- and school-aged children (Halle and Darling-Churchill, 2016; Humphrey et al., 2011; Martinez-Yarza et al., 2023). They have identified a clear need for further assessment tools in the area of SEL, particularly at the start of school attendance (McKown, 2017; for German instruments assessing emotional competence see Wiedebusch and Petermann, 2006). Halle and Darling-Churchill (2016) reviewed 75 instruments for measuring social–emotional competence and identified four common domains that are often assessed: (1) social competence, (2) emotional competence, (3) behavior problems and (4) self-regulation. Since social–emotional competence encompasses various aspects, the definition of specific skills is helpful for its assessment. While competence is a broad term, skills are described as specific behaviors used to competently complete a task (Gresham, 1986). Based on Denham et al., (2014), we identified following skills as being important for measuring social–emotional competence: (1) relational/prosocial skills and (2) emotional competence skills. As found by Halle and Darling-Churchill (2016), we also included (3) behavior problems. We now focus on these three areas in more detail.

#### 1.1.1 Relational/prosocial skills

“Prosocial behavior is defined as any voluntary, intentional action that produces a positive or beneficial outcome for the recipient regardless of whether that action is costly to the donor, neutral in its impact, or beneficial” (Grusec et al., 2002, p. 458). Measuring prosocial behavior via questionnaires, respondents are asked how often they have performed certain prosocial behaviors (referred to as prosocial responding), such as comforting or helping others (Eisenberg and Mussen, 2009). One important relational skill is social problem solving, which involves adapting your behavior to meet the specific social demands of each situation (Kanning, 2002). As “children’s social behaviors are best understood as responses to specific situations or tasks” (Dodge et al., 1985, p. 351) making use of psychological testing is not deemed appropriate. Hence, performance measures and Situational Judgement Tests (SJTs) are viewed as more promising in assessing social–emotional skills (Soto et al., 2021). In SJTs, a hypothetical situation is presented with possible reactions or responses. The participant has to rank or rate the responses. In developing SJTs, different options concerning scenarios and responses are possible. Typically, a specific situation is described and a few possible reactions to the situation are offered. Usually, the participants have to choose the reaction they are most likely to show (McDaniel and Nguyen, 2001). In measuring social–emotional skills, the perceived validity and robustness of SJT results make them preferable to self-report measures (Abrahams et al., 2019). Murano et al. (2021) developed and validated a SJT for primary school children (third and fourth grade), using a five-point scale to rate each response to the situations presented. While the subscales Grit and Teamwork were found to be reliable in a SJT (*α* = 0.80 and 0.76), three other subscales, i.e., Resilience, Curiosity and Leadership were found to have low reliability (*α* < 0.60). For older students, the FEPAA (Fragebogen zur Erfassung von Empathie, Prosozialität, Aggressionsbereitschaft und aggressivem Verhalten: Lukesch, 2006) is an instrument frequently used in German speaking countries. This questionnaire is used for teenagers (12–16 years) and assesses empathy, prosocial behavior and aggressive behavior reliably (*α* = 0.75), also using a SJT approach.

#### 1.1.2 Emotional competence skills

Key components of emotional competence are emotion knowledge, emotion utilization and emotion regulation (ER). Emotion knowledge refers to understanding expressions and feelings. Emotion utilization is the adaptive use of the deployment of emotion arousal (Izard et al., 2011). The process of ER describes how individuals can influence what emotions they experience as well as when and how they express them (Gross, 1998). It “is the neural, cognitive, and behavioral/action processes that sustain, amplify, or attenuate emotion arousal and the associated feeling/motivational, cognitive, and action tendencies” (Izard et al., 2011, p. 45). Especially for school- aged children, acquiring ER is important because “managing how and when to show emotion becomes crucial” (Denham et al., 2002, p. 309) in social situations. In the same context, Saarni et al. (2006) also talk about emotion management. There are five sets of emotion regulatory processes: (1) situation selection, (2) situation modification, (3) attention deployment, (4) cognitive change, and (5) response modulation (Gross, 1998, 2015). One instrument for assessing different ER strategies is the FEEL-KJ (Fragebogen zur Erhebung der Emotionsregulation bei Kindern und Jugendlichen: Grob and Smolenski, 2005). This instrument, for children and adolescents aged 10–20 years, measures 15 ER strategies for three different emotions (anger, sadness, anxiety). It has been used and validated in German speaking countries (Grob and Smolenski, 2005) and the Netherlands (Cracco et al., 2015). While showing good reliability for this age group (*α* = 0.69–0.91), it has not been used with younger children. Another German instrument used in measuring emotional skills is the EMO-KJ (Diagnostik- und Therapieverfahren zum Zugang von Emotionen bei Kindern und Jugendlichen: Kupper and Rohrmann, 2018). It measures emotional differentiation and situational behavior in children aged 5–16. The EMO-KJ is only used in a one-to-one setting and in the manual does not contain any information on the reliability of the instrument.

#### 1.1.3 Behavior problems

When describing and measuring broad difficulties in behavioral, emotional and social areas, it is common to distinguish between internalizing and externalizing behavior problems (Achenbach et al., 2016). Children with externalizing problems are likely to exhibit poor self-control, or are hyperactive, and often score high on anger and aggressive behavior (Liu, 2004). Children with internalizing problems are prone to sadness, may be anxious, and may show signs of depression (Liu et al., 2011). Both externalizing and internalizing behavior problems are correlated with lower self-regulation (Yavuz-Müren et al., 2022; Eisenberg et al., 2001). Bornstein et al. (2010) found lower social competence at the age of four leading to more externalizing and internalizing behavior at the age of 10 and to more externalizing behavior at the age of 14. Even though there is an association between behavior problems and social–emotional competence, it is important to note that “not all children with problem behavior are socially unskilled, or vice versa” (Hukkelberg and Andersson, 2023, p. 2). Also, lower skills in ER have been shown to be associated with behavioral problems (Aldao et al., 2016; Kullik and Petermann, 2013). Children with externalizing behavior problems exhibited higher anger levels than children not showing behavior problems. Unregulated anger might be one reason for these children to exhibit externalizing behavior problems (Eisenberg et al., 2001). In addition to internalizing and externalizing behavior, some scholars also differentiate children in terms of social withdrawal. Such children are at risk of developing internalizing problems and of deficiencies in problem-solving skills due to their lack of social interactions (Rubin et al., 1991; Rubin and Chronis-Tuscano, 2021; Boivin et al., 1995).

Sources of information in assessing internalizing and externalizing behaviors in children may be peers, parents, teachers or the children themselves. There are several measures that focus on parent or teacher reports, such as the Strength and Difficulties Questionnaire (SDQ, Goodman, 1999) and the Child Behavior Checklist (CBCL, Döpfner et al., 2014). Results in parent and teacher reports may differ as a result of the different contexts and perspectives entailed, especially when rating internalizing behavior (Gresham et al., 2010; Navarro et al., 2020). An investigation into the cross-informant agreement found that parent-teacher informants showed a stronger association (*r* = 0.33) in comparison to student-teacher informants (*r* = 0.23). A comparison of the ratings provided by two teachers resulted in an observed agreement of *r* = 0.63. This indicates that even in the same context, teachers may exhibit distinct observational behaviors (Gresham et al., 2018). In addition, some studies have shown rather low correlations between teacher and student ratings when assessing externalizing and internalizing behaviors (Huber et al., 2019) and social–emotional skills (Mudarra et al., 2022). With respect to internalizing behavior, the child’s perspective is particularly important owing to teachers’ tendencies to underestimate internalizing behaviors in their own students (Huber et al., 2019). Neil and Smith (2017) found small positive associations between children’s and teachers’ reports of anxiety (*r* = 0.14) and concluded that teachers only have limited sensitivity in recognizing anxiety symptoms in children. As school children are quite capable of offering very reflective insights concerning their own situation (Wigelsworth et al., 2010), adoption of a multi-informant perspective is recommended in order to obtain a comprehensive picture (e.g., Abrahams et al., 2019).

#### 1.1.4 Existing instruments measuring social–emotional competence

Existing instruments measuring social–emotional competence in children are limited, particularly in the German language context. For example, some German instruments, such as EMO-KJ and FEEL-KJ, assess specific aspects of emotional competence, but tools measuring multiple facets of social–emotional competence are less common. One relatively new instrument for English speaking children is SELweb (McKown, 2019). It is a web-based, self-administered direct assessment battery of social–emotional comprehension with five modules. It measures emotion recognition, social perspective-taking, social problem-solving, self-control - delay of gratification, self-control - frustration tolerance. Using a sample of 4,419 children in the United States, the results at factor level showed sufficiently high internal consistency (*r*_yy_ = 0.79–0.88) and temporal stability (*r*_12_ = 0.55–0.79) at factor score level and the four-factor model was confirmed (McKown, 2019).

### 1.2 The current study

Although several instruments are available for measuring different aspects of social–emotional skills, our literature review did not identify any suitable German-language instruments that (1) are designed especially for children in the early school years, (2) can be used in group settings, and (3) assess multiple dimensions of social–emotional skills. Addressing this gap, we developed the Grazer Screening Instrument to Assess Social–Emotional Skills (GraSEF). The development of GraSEF was based on Denham’s model of social–emotional competences and further informed by the findings of Halle and Darling-Churchill (2016) integrating behavior difficulties. GraSEF includes five subtests to focus on (1) *Behavior in Social Situations,* (2) *Prosocial Behavior*, (3) *Emotion Regulation Strategies,* (4) *Emotion Recognition,* and on *(5) Self-Perception of Emotions* (as displayed in Figure 1). The subtest *Behavior in Social Situations* is a SJT approach, similar to that used in Murano et al. (2021), and focuses on *Internalizing Behavior*, *Externalizing Behavior*, *Prosocial Behavior* and *Social Withdrawal*. This design ensures that GraSEF captures a broad range of social–emotional skills that are critical for childhood development.

The target group is children aged between 6 and 8. Given the various advantages of digital tools in such a context (e.g., concerning student motivation, ease of evaluation; see for example Blumenthal and Blumenthal, 2020), we decided to use tablets instead of print surveys. We provided the children with headphones so that they could hear all questions, items, and instructions while also seeing them in written form. Thus, no reading skills were necessary to complete the GraSEF, and each child could work at his/her own pace.

The purpose of the GraSEF is to provide reliable measurement of social–emotional skills while also ensuring easy classroom implementation and maintaining high motivation in the children. The present study aims to investigate its user-friendliness for group settings in second grade inclusive classrooms (also taking account of those students with low reading skills), and to evaluate the screening instrument’s psychometric quality and item characteristics. The following research questions are addressed:

1. What do the first item analyses reveal regarding distribution, discriminatory power, and item difficulty?
2. How well does the proposed test structure match the results of the factor analysis?
3. Do the screening instrument’s subscales meet the psychometric quality criteria, specifically regarding reliability and validity?
4. How user-friendly is the assessment for second graders in inclusive classroom settings, and which adjustments are needed to enhance its usability?

In addition to these research questions, we conducted exploratory analyses of gender differences in the subtest *Behavior in Social Situations* and explored the following question:

1. Are there any gender differences in the different subscales of the subtest Behavior in Social Situations?

We expected the subtest 1 (*Behavior in Social Situations*) to have four different factors (externalizing behavior, internalizing behavior, problem-solving/assertive behavior, social withdrawal) and subtest 2 (*Emotion Regulation Strategies*) to be three-factorial. All other subtests ((3) *Emotion Regulation*, (4) *Emotion Recognition* and (5) *Self-Perception of Emotions*) were expected to be one-factorial. Furthermore, we expected the scores of the subtest *Behavior in Social Situations* to correlate positively with the respective teacher rating. The pre-registration of this study (March, 19th, 2024) is available on https://doi.org/10.17605/OSF.IO/6MVHP.

## 2 Methods

### 2.1 Participants

The sample consisted of 68 students from six Grade 2 classrooms in four schools (age: *M* = 8.23, *SD* = 0.48). About half of them were female (48.06%) and 52.94% spoke German as their family language. A total of 75 students participated, but seven were excluded due to inadequate proficiency in German (2 points on a 5-point teacher rating scale and an “a.o.-status”^1^). The participating schools were located in urban and suburban areas. The sample includes a significant proportion of children with diverse cultural backgrounds, for example indicated by the percentage of children with a family language other than German. As this is a pilot study with preliminary analyses of the GraSEF, we aimed for a sample size of 60 to get a good estimate of item distribution and internal consistency. Methodological studies have shown that 30 participants are sufficient for scale development (Johanson and Brooks, 2009).

Parental consent was obtained for all participants. All students included in the study also gave their consent before starting the screening procedure. The study was approved by the ethics committee of the University of Graz and the Styrian Board of Education.

### 2.2 Instruments

#### 2.2.1 GraSEF—screening to assess social–emotional skills

The screening’s five subtests were administered via an online survey tool (Limesurvey, n.d., Version 3.28.22) and modified for the target group (graphic design, font type and font size, audios to guide the children through the test). Before testing the items quantitatively, we piloted them in a sample of 10 students (age: *M* = 8.10, *SD* = 0.45; 40% female; 50% German as a first language) in individual settings. We used screencasts and think-aloud protocols to find out more about usability and comprehensibility. The piloting was also used to investigated how well the students could relate to the situations in the subtest *Behavior in Social Situations*. After this pilot study, we revisited the items and improved the guidance and navigation through LimeSurvey. Based on students’ feedback, we implemented several revisions to enhance item clarity. For example, in one situation, a pen was falling on the floor, but students indicated that they were not yet using pens for writing. This feedback helped us to revise the situations to better align with the students’ experiences. We also changed one pictures of the subtest *Behavior in Social Situations*. To finalize the items for each subtest, we conducted face validity checks, ensuring that each item aligned with the intended constructs. In particular, for the subtest *Behavior in Social Situations*, we analyzed all the items without the situational prompts to confirm comparability and similarity within each subscale. This process ensured, that the final itemset was both meaningful to the students and suitable for measuring specific skills.

For each subtest, we used a 5-point Likert scale with the word-based response format rating of ‘never’ to ‘very often’ or ‘no’ to ‘yes’ in order to achieve better scale properties and more discriminating results than normally possible with the traditional dichotomous yes-no format (Mellor and Moore, 2014). The resulting screening instrument consisted of five subtests and a total of 112 items. All subtests and items can be found as Supplementary material A. Within the subtests, we randomized the item order.

##### 2.2.1.1 Subtest 1: behavior in social situations

**Figure 2:**
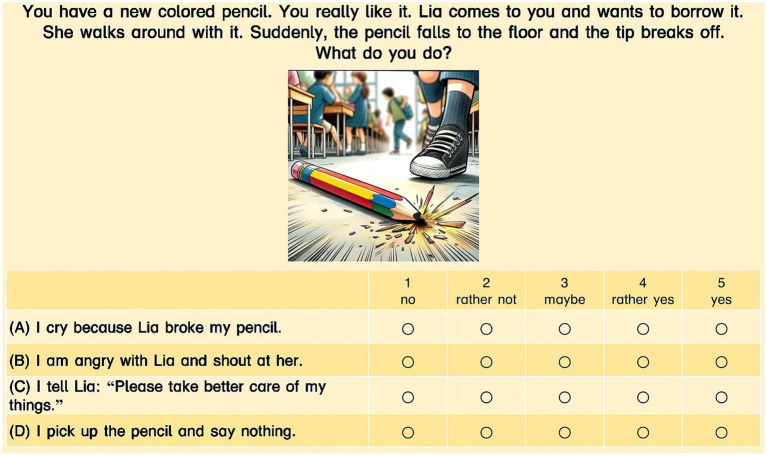
Situation 6 (SV6) in the Subtest *Behavior in Social Situations* and the different reaction options. (A) Internalizing Behavior (SV6_int: “I cry because Lisa broke my pencil.”); (B) Externalizing Behavior (SV6_ext: “I am angry with Lia and shout at her.”); (C) Problem-Solving/Assertive Behavior (SV6_pro: “I tell Lia: Please take better care of my things.”); (D) Social Withdrawal (SV6_su: “I pick up the pencil and say nothing.”). During the digital screening procedure, the items are presented to the children in random order.

The first subtest was an SJT and contained 15 challenging everyday situations in schools (e.g., feeling left out). To make the situations more accessible for young children, we generated pictures for each situation, using the Artificial Intelligence-Tool Dall-E via ChatGPT-4 (OpenAI, 2023). Using an approach similar to Murano et al. (2021), students were asked to decide, based on a five-point Likert scale (‘1-no’, ‘2-rather not’, ‘3-maybe’, ‘4-rather yes’, ‘5-yes’), how likely they were to react as described in the respective option. There were four reaction options for each situation (e.g., for the situation 6 (SV6), there were four items SV6_int, SV6_ext, SV6_pro, SV6_su, see Figure 2). Each reaction option represented one subscale, making a total of 60 items (15 for each subscale). The following four subscales were covered:

- *Internalizing Behavior:* This subscale included anxious and/or depressive behavior (Eisenberg et al., 2001; Liu et al., 2011), mainly being sad or anxious. (SV6_int: I cry because Lia broke my pencil.)
- *Externalizing Behavior:* This subscale included reactions that show anger, aggression and/or hyperactivity (Liu, 2004). (SV6_ext: I am angry with Lia and shout at her.)
- *Problem-Solving/Assertive Behavior:* For this subscale, we defined behavior as proactive if the student was able to express his/her needs and to tell the other student what he or she wanted. Initially (see pre-registration), we named the scale “prosocial behavior,” but after reviewing and revisiting some of the items we decided that the term “problem-solving/assertive behavior” was more appropriate to describe the subscale’s content. (SV6_pro: I say to Lia: “Please take better care of my things.”)
- *Social Withdrawal:* This subscale included “all forms of solitary behavior when encountering familiar and/or unfamiliar peers. Simply put, social withdrawal is construed as isolating oneself from the peer group” (Rubin et al., 2002, p. 330) and not having adequate behavior strategies to deal with a certain social situation. (SV6_su: I pick up the pencil and say nothing.)

Before creating suitable items for the four subscales of this subtest, we developed 15 scenarios relating to challenging situations for school-aged children. In generating these situations, we reviewed existing instruments, such as the FEPAA (Lukesch, 2006) and the TOPS (Taxonomy of Problematic Social Situations for Children: Dodge et al., 1985). Martín-Antón et al. (2016) used TOPS to examine the most difficult situations for children experiencing rejection in the first school year. They found that (1) being disadvantaged, (2) respecting authority and rules, (3) responding to their own success, and (4) showing prosocial and empathic behaviors are the most difficult situations for these children. Additional considerations for creating the situations were used by Cillessen and Bellmore (2002), who presented four important social tasks for school-aged children: (1) conflict resolution, (2) peer group entry, (3) competent play with others and (4) emotion regulation. Merging these findings, we generated at least two situations for each of the following scenarios: someone taking belongings away (SV1, SV2), social exclusion (SV3, SV4), damaging property (SV5, SV6), joining a peer group (SV7, SV8), being teased by others (SV9, SV10, SV11), social assertiveness (SV12, SV13) and dealing with other people’s mistakes (SV14, SV15). To minimize name bias (Nick, 2017), we used short and neutral names for the protagonists of the situations (e.g., Lia, Rob, Tom).

##### 2.2.1.2 Subtest 2: prosocial behavior

For measuring prosocial behavior, we used a questionnaire measure of prosocial response based on Gasteiger-Klicpera et al. (2006). Students are asked how often they have enacted certain prosocial behaviors in the classroom during the previous two weeks (e.g., “How often have you helped other children in the class?”). The subtest consisted of five items and the students had to provide ratings from 1 to 5 (‘1-never’, ‘2-rarely’, ‘3-sometimes’, ‘4-often’, ‘5-very often’).

##### 2.2.1.3 Subtest 3: emotion regulation strategies (ERS)

We asked the students what they did to manage their emotions and, like Grob and Smolenski (2005), we distinguished between three different basic emotional dimensions (anger, sadness, anxiety). Students had to indicate on a five-point Likert scale how often they used certain ERS (‘1-never’, ‘2-rarely’, ‘3-sometimes’, ‘4-often’, ‘5-very often’). The following possible strategies were investigated (‘To make myself less angry/sad/afraid, …’): (1) cognitive distraction (four items; e.g., ‘…, I think of nice things.’), (2) cognitive reappraisal (three items; e.g., ‘…, I tell myself it’s not that bad.’), (3) problem-solving (two items; e.g., ‘…, I try to change what makes me angry/sad/afraid.’) and (4) maladaptive – meaning having no strategy (two items; e.g., ‘I do not know what to do, to make myself less angry/sad/afraid.’). For each emotional dimension (anger, sadness, anxiety), there were a total of 11 items.

##### 2.2.1.4 Subtest 4: emotion recognition

To assess emotion recognition, we employed a common approach using images. There are universal facial expressions for happiness, anger, sadness, disgust and fear (Ekmann, 1992). In this subtest, children are presented with an image of a child’s facial expressions and have to choose the appropriate emotion from the five basic emotions presented (happiness, sadness, anger, fear and surprise). The 10 images show a boy or a girl with one basic emotional expression. To obtain the image set for this subtest, 40 images were generated by artificial intelligence (Playground AI, 2024) and pre-evaluated twice by master’s and doctoral students (*N*_first pre-evaluation_ = 20, *N*_second pre-evaluation_ = 14). All of the items showed at least 80% agreement in pre-evaluation, except for one item (sad boy) with an agreement level of 71.4%. As generating an appropriate image for disgust proved to be extremely difficult, we excluded this from the analyses. When selecting and generating the images, we also took various aspects of diversity into account (e.g., children with glasses, different hair and skin colors, …).

##### 2.2.1.5 Subtest 5: self-perception of emotions (initially called emotion perception)

In this subtest, there are four sentences, for each of which the children have to indicate one emotion out of the three available (e.g., ‘How do you feel when you get a great present?’). For each situation, there is one most suitable emotion. We developed four sentences suitable for representing the emotions happiness, anger and sadness.

#### 2.2.2 Reading self-concept and reading interest

Additionally, children’s *Reading Self-Concept* and *Reading Interest* was assessed using 10 items. These items were modified based on McElvany et al. (2008) and Rauer and Schuck (2003). Participants used ratings from 1 to 5 (‘1-never’, ‘2-rarely’, ‘3-sometimes’, ‘4-often’, ‘5-very often’), to indicate whether they agreed with a particular statement (e.g., *Reading Self-Concept:* ‘I am a good reader’, *Reading Interest:* ‘I like reading’). While this scale is not analyzed further in the present paper, it was part of the screening procedure and the preregistration.

#### 2.2.3 Teacher short questionnaire

This self-constructed questionnaire was designed to obtain basic information on the children participating (age, gender, first language(s)) and was filled out by teachers. In addition, German language skills and the following five aspects of the children’s social–emotional skills were rated by the teachers from ‘1—low-skilled’ to ‘5—highly skilled’: (a) emotion regulation skills, (b) prosocial behavior (e.g., helpfulness, cooperation), (c) externalizing behavior (e.g., aggression, hyperactivity), (d) internalizing behavior (e.g., depressive behavior, sadness), and (e) social withdrawal and shyness.

#### 2.2.4 Transcripts and observation logs

The children were observed while completing the screening procedure and we noted any related difficulties or relevant questions. After completing the GraSEF, an additional question was used to ascertain their feelings towards the test (“Did you like the questions?”), and was answered using a five-point Likert scale (‘1-no’ to ‘5-yes’). In addition, we asked the children verbally what was difficult or easy, and also what they liked best or least. The comments were then written down.

### 2.3 Procedure

This study was conducted in schools in Styria, a province of Austria. Small groups (4 to 8 students), accompanied by one to three researchers or master’s students, completed the screening instrument in quiet rooms. Each child was provided with a tablet and headphones. After a brief introduction and explanation, each participant was asked to turn on the tablet, put on the headphones, and to start the screening activity. All instructions and items had been previously recorded and LimeSurvey automatically played the appropriate audio as the participants moved from item to item. It was also possible to replay the audios. Children had to complete each question before continuing with the next question. After completing questions regarding *Reading Interest/Reading Self-Concept*, the subtests of the GraSEF were then presented. We observed the children and ensured that each participant received support when needed. After completing the GraSEF, we asked the participants a few questions about their interest in the questions presented and what they liked most/least about the questions. The teachers completed the teacher short questionnaire in advance.

### 2.4 Data analysis

Data analysis was performed using R statistical software (Version 4.2.1, R Core Team, 2023) and RStudio (Posit team, 2024). Before starting the analysis, we recoded all items from 1–5 to 0–4. For data cleaning and recoding, descriptive analyses and the initial item analyses (discriminatory power, difficulty, mean and standard deviation), we used the packages tidyverse (Wickham et al., 2019), psych (Revelle, 2024) and car (Fox and Weisberg, 2019). Each item was analyzed graphically using boxplots and histograms. We also checked whether the data was normally distributed using the Shapiro–Wilk test and also looked at its descriptive properties (skew, kurtosis). Since students were required to respond to all questions, there was no missing data.

We also checked the psychometric quality criteria, i.e., reliability and validity. McDonald’s Omega (*ω*) and Cronbach’s Alpha (*α*) were used to measure the internal consistency of each scale (package MBESS, Kelly, 2023). We report McDonald’s ω and Chronbach’s α to get a better understanding of GraSEF’s reliability and to allow for comparisons between both measures. Using ω alongside confidence intervals (CI) is increasingly recommended as it relies on fewer assumptions about the data than Chronbach’s α and provides a more accurate estimation of internal consistency (Dunn et al., 2014). For the scales *Emotion Recognition* and *Emotion Perceptions*, the Kuder–Richardson Formula 20 (KR-20; package validate; Desjardins, 2024), which is suitable for true/false answers, was used. For the initial validity check, we analyzed construct validity (convergent validity) using teacher ratings. Correlation analyses were used to assess the association between the teacher ratings and the student responses, as well as the correlation within each scale. To account for non-normally distributed data, Spearman’s rho (*ρ*) was used to calculate correlations.

To assess the factorial structure of each scale, we conducted confirmatory factorial analyses (CFA). We used the packages lavaan (Rosseel, 2012) and semplot (Epskamp, 2022). Owing to the small sample size, we simplified the models as much as possible and used the WLSMV (Weighted Least Squares Mean and Variance adjusted) estimator for the analysis. The WLSMV estimator is designed for use with ordinal data, such as ordered rating scales (Muthén and Curran, 1997). We hypothesized a four-dimensional model for *Behavior in Social Situations* and a three-dimensional model for *ERS*. We also evaluated the one-dimensionality of the other three subscales. We used following Goodness-of-Fit Indices for evaluating the results of the CFA: Chi-Square (χ^2^) goodness of fit test and its degrees of freedom, the Comparative Fit Index (CFI), the Root Mean Square Error of Approximation (RMSEA) with its 95% CI and the Standardizes Root Mean Square Residual (SRMR). Following the recommendation of Hu and Bentler (1999) and Moosbrugger and Kelava (2020), the cut-off criteria applied were: CFI > 0.95, RMSEA < 0.08, SRMR < 0.10. Given the limited sample size, we did not interpret the results, but we do present them in the form of an overview. For the one-dimensional tests, we assumed a tau-congeneric model.

To examine the gender difference in the subscale *Behavior in Social Situations* we calculated a MANOVA followed post-hoc ANOVAs. This analysis was performed using IBM SPSS Statistics (IBM Corp, 2020).

## 3 Results

### 3.1 Initial item analyses

#### 3.1.1 Behavior in social situations

The items of this subtest were assigned to four subscales (*Internalizing Behavior, Externalizing Behavior, Problem-Solving/Assertive Behavior* and *Social Withdrawal*) and for each subscale, the sum was calculated. The difficulty of the items varied across the four subscales and ranged from 0.29 to 0.56 in the subscale *Internalizing Behavior*, from 0.12 to 0.47 in the subscale *Externalizing Behavior*, from 0.75 to 0.89 in the subscale *Problem-Solving/Assertive Behavior*, and from 0.14 to 0.67 in the subscale *Social Withdrawal*. For more descriptive data, see Supplementary material B. In order to shorten the screening process, situations that tended to impair comprehension or exhibited insufficient item distribution were identified and removed from the subtest. Items SV3_ext and SV5_su showed low difficulty and high kurtosis, items SV2_pro, SV5_pro and SV11_pro showed insufficient distribution (skew > |2|) and item SV9_su showed low discriminatory power (*r*_i(t-i)_ = 0.16). We thus decided to exclude the situations 2, 5 and 9 and to modify some of the items (e.g., SV3_ext and SV11_pro). Items in the subscale *Problem-solving/Assertive Behavior* were changed to eliminate the possibility of providing socially desirable responses. The Shapiro–Wilk test showed that the sum values were not normally distributed, except for the Subscale *Social Withdrawal* (*W* = 0.99, *p* = 0.727). The subscales *Internalizing Behavior* and *Externalizing Behavior* were right-skewed and the subscale *Problem-Solving/Assertive Behavior* was left-skewed. The sum scores of the final subscales are presented in Table 1, with a maximum value of 48 being found for the subscale *Problem-solving/Assertive Behavior*. The highest mean was attained for the subscale *Problem-Solving/Assertive Behavior* (*M* = 38.79, *SD* = 7.63) and the lowest for *Externalizing Behavior* (*M* = 13.29, *SD* = 10.58).

**Table 1:** Subscale parameters.

Subscale	*M*	SD	Min	Max	Skew	Kurtosis	ω^a^	95% CI	α	95% CI	KR-20
Internalizing behavior (12 items)	17.24	12.43	0	42	0.26	−0.78	0.86	0.79–0.90	0.86	0.80–0.90	
Externalizing behavior (12 items)	13.29	10.58	0	44	0.96	0.43	0.83	0.75–0.89	0.83	0.76–0.88	
Problem-solving/assertive behavior (12 items)	38.79	7.63	4	48	−1.69	5.03	0.72	0.42–0.87	0.72	0.61–0.81	
Social withdrawal (12 items)	23.41	9.27	1	43	−0.18	−0.33	0.73	0.59–0.82	0.72	0.61–0.81	
Prosocial behavior (5 items)	2.81	0.76	0.4	4	−0.62	0.47	0.71	0.54–0.82	0.71	0.58–0.80	
ERS anger (9 items)	2.29	0.98	0	3.89	−0.60	−0.44	0.88	0.81–0.92	0.87	0.82–0.91	
ERS sadness (9 items)	2.26	0.97	0	4	−0.31	−0.41	0.88	0.81–0.92	0.88	0.83–0.92	
ERS anxiety (9 items)	2.23	0.99	0	4	−0.26	−0.51	0.86	0.78–0.91	0.86	0.81–0.91	
Emotion recognition (9 items)	8.03	1.12	3	9	−1.75	3.95			0.50	0.30–0.66	0.51
Self-perception of emotions (3 items)	3.50	0.81	1	4	−1.31	1.16			0.32	−0.02–0.56	0.36

ω, McDonald’s omega; α, Cronbach’s alpha; KR-20, Kuder–Richardson Formula 20; ERS, emotion regulation strategies; 95% CI, 95% confidence interval. ^a^ McDonald’s Omega (ω) was calculated with bias-corrected and accelerated (Bca) bootstrap confidence intervals (Number of Bootstrap Resamples = 1,000).

#### 3.1.2 Prosocial behavior

The item difficulty of the five items ranged from *P*_i_ = 0.58 to *P*_i_ = 0.77. The distribution and other item characteristics were good and within the proposed range of between 0.20 and 0.80. On the scale from 0 to 4, the students had an average score of 2.81 (*SD* = 0.76).

#### 3.1.3 Emotion regulation strategies

Here, item difficulty ranged from *P*_i_ = 0.48 to *P*_i_ = 0.70. Almost all items of the subtest showed sufficient distribution. However, the inverted items describing maladaptive strategies (W10, W11, T10, T11, A10, A11) showed very low or negative discriminatory power for each emotional dimension. These were excluded from further analysis, thus resulting in nine items per subscale.

#### 3.1.4 Emotion recognition

The item difficulty ranged from *P*_i_ = 0.59 (image7, anxious boy) to *P*_i_ = 0.99 (image5, angry boy). On average, the students solved 8.62 (*SD* = 1.35, *Min* = 3, *Max* = 10) out of 10 picture tasks correctly. Because of the low consensus and discriminatory power of item image7, we removed it from further analyses.

#### 3.1.5 Self-perception of emotions

The item difficulty ranged from *P*_i_ = 0.75 (E1) to *P*_i_ = 0.99 (E2). On average, the students answered 3.40 (*SD* = 0.81, *Min* = 1, *Max* = 4) out of 4 sentences correctly. Item E2 (‘How do you feel, when you receive a great gift?’) was excluded from further analyses due to its difficulty. Table 1 shows the mean and other scale characteristics of the subscales.

### 3.2 Factorial structure of the subtests and subscales

For the subtest *Behavior in Social Situations*, we tested a four-factorial model with all items. This initial model yielded χ^2^_WLSMV_ (1074) = 1539.93, *p* < 0.001, CFI = 0.915, RMSEA = 0.080 (95% CI [0.071, 0.089]), SRMR = 0.150. Factor loadings ranged from 0.297 to 0.899 with two loadings not being statistically significant (SV6_su: *λ* = 0.169, *p* = 0.168, and SV13_su: *λ* = 0.137, *p* = 0.262). Consequently, we removed these items and re-tested the model, which resulted in *χ^2^*_WLSMV_ (983) = 1367.17, *p* < 0.001, CFI = 0.929, RMSEA = 0.076 (95% CI [0.066, 0.086]), SRMR = 0.148. The covariances between the latent variables ranged from 0.17 (internalizing behavior/problem-solving, assertive behavior) to 0.78 (internalizing behavior/social withdrawal). The revised model was not fully consistent with the fit indices preregistered in the online preregistration. We additionally checked a one-factorial model with all items of the subtest *Behavior in Social Situations*. This yielded χ^2^_WLSMV_ (1080) = 2247.46, *p* < 0.001, CFI = 0.788, RMSEA = 0.127 (95% CI [0.120, 0.134]), SRMR = 0.181 (Table 2).

**Table 2:** CFA comparing factor models.

Subtest	Factors	χ^2^_WLSMV_(df)	*p*	CFI	RMSEA	SRMR	*λ* min/*λ* max
Behavior in social situations ^a^	4	1367.17 (983)	<0.001	0.929	0.076 (0.066–0.086)	0.148	0.297/0.899
Behavior in social situations	1	2247.46 (1080)	<0.001	0.788	0.127 (0.120–0.134)	0.181	0.094/0.807
ERS	3	358.24 (321)	=0.075	0.996	0.042 (0.000–0.064)	0.096	0.351/0.752
ERS	1	398.16 (299)	<0.001	0.988	0.070 (0.051–0.088)	0.105	0.290/0.692

ERS, emotion regulation strategies; χ^2^, Chi^2^; df, degrees of freedom; CFI, comparative fit index; RMSEA, root mean square error of approximation; SRMR, standardizes root mean square residual; λ min, minimal factor loading; λ max, maximum factor loading. ^a^ After removing non-significant variables (SV6_su, SV13_su).

For the subtest *ERS,* we proposed a three-factorial model, *χ*^2^_WLSMV_ (321) = 358.24, *p* = 0.075, CFI = 0.996, RMSEA = 0.042 (95% CI [0.000, 0.064]), SRMR = 0.096. In comparison, the one-factor model showed a poorer fit, *χ*^2^_WLSMV_ (299) = 398.16, *p* < 0.001, CFI = 0.988, RMSEA = 0.070 (95% CI [0.051, 0.088]), SRMR = 0.105.

Table 3 shows the CFA results checking one-dimensionality for all subscales except for *Self-Perception of Emotions*, for which no model could be identified. Due to the small sample size, the analysis of the factorial structure of the GraSEF should be interpreted with caution and shows only a first insight.

**Table 3:** CFA for all subscales.

Subscale	Items	Factors	χ^2^_WLSMV_(df)	*p*	CFI	RMSEA	SRMR	*λ* min/*λ* max
Internalizing behavior	12	1	63.38 (54)	0.179	0.993	0.051 (0.000–0.096)	0.098	0.493/0.805
Externalizing behavior	12	1	44.80 (54)	0.810	1.000	0.000 (0.000–0.051)	0.101	0.357/0.784
Problem-solving/assertive behavior	12	1	46.03 (54)	0.771	1.000	0.000 (0.000–0.055)	0.111	0.265/0.747
Social withdrawal	12	1	90.17 (54)	0.001	0.890	0.100 (0.062–0.135)	0.130	0.235/0.763
Prosocial behavior	5	1	2.79 (5)	0.733	1.000	0.000 (0.000–0.012)	0.042	0.492/0.792
ERS: anger	9	1	33.34 (27)	0.186	0.995	0.059 (0.000–0.118)	0.079	0.618/0.808
ERS: sadness	9	1	36.57 (27)	0.103	0.993	0.073 (0.000–0.128)	0.082	0.692/0.812
ERS: anxiety	9	1	18.20 (27)	0.897	1.000	0.000 (0.000–0.043)	0.065	0.594/0.775
Emotion recognition	9	1	20.00 (27)	0.831	1.000	0.000 (0.000–0.058)	0.115	0.301/0.986

ERS, emotion regulation strategies; χ^2^, Chi^2^; df, degrees of freedom; CFI, comparative fit index; RMSEA, root mean square error of approximation; SRMR, standardizes root mean square residual; λ min, minimal factor loading; λ max, maximum factor loading.

### 3.3 GraSEF’s reliability

The internal consistency of the scales ranged from *ω* = 0.71 to *ω* = 0.88. All subscales, except *Emotion Recognition* and *Emotion Perception*, met the preregistered thresholds (*ω*/*α* > 0.70) and demonstrated acceptable to good internal consistency. Looking at the 95% CI, the scale *Problem-Solving/Assertive Behavior* showed a wide CI, indicating less precision in the estimation. For the subscales *Emotion Recognition* and *Emotion Perception*, the internal consistency was rather low (*KR-20* = 0.52 and 0.36). Table 1 shows the subscale’s parameters and its internal consistencies with CI.

Table 4 shows the correlations between the scales. There is a medium correlation between *Internalizing Behavior* and *Social Withdrawal* (*ρ* = 0.49**) and *Internalizing Behavior* and *Externalizing Behavior* (*ρ* = 0.46**). The higher the scores in *Internalizing Behavior*, the higher they were for *Externalizing Behavior* and *Social Withdrawal*. There was no correlation between *Internalizing Behavior* and *Problem-Solving/Assertive Behavior*, but a negative correlation was found between *Externalizing Behavior* and *Problem-Solving/Assertive Behavior*. We also found a strong correlation between *ERS* anger and sadness (*ρ* = 0.80**) and sadness and anxiety (*ρ* = 0.70**), showing that *ERS* for the three emotional dimensions display similarities.

**Table 4:** Correlations between the subscales.

	1	2	3	4	5	6	7	8	9
1 Internalizing behavior	–								
2 Externalizing behavior	*ρ* = 0.46^**^	–							
3 Problem-solving/assertive behavior	*ρ* = 0.09	*ρ* = −0.40^**^	–						
4 Social withdrawal	*ρ* = 0.49^**^	*ρ* = 0.33^*^	*ρ* = 0.19	–					
5 Prosocial behavior	*ρ* = −0.05	*ρ* = 0.04	*ρ* = 0.16	*ρ* = −0.07	–				
6 ERS: anger	*ρ* = 0.13	*ρ* = 0.04	*ρ* = 0.28^*^	*ρ* = 0.28^*^	*ρ* = 0.40^**^	–			
7 ERS: sadness	*ρ* = 0.16	*ρ* = 0.09	*ρ* = 0.23	*ρ* = 0.18	*ρ* = 0.33^*^	*ρ* = 0.80^**^	–		
8 ERS: anxiety	*ρ* = 0.11	*ρ* = −0.03	*ρ* = 0.31^*^	*ρ* = 0.12	*ρ* = 0.38^**^	*ρ* = 0.58^**^	*ρ* = 0.70^**^	–	
9 Emotion recognition	*ρ* = 0.10	*ρ* = −0.16	*ρ* = 0.03	*ρ* = −0.04	*ρ* = −0.02	*ρ* = 0.10	*ρ* = 0.00	*ρ* = −0.03	–
10 Self-perception of emotions	*ρ* = 0.20	*ρ* = −0.10	*ρ* = 0.22	*ρ* = −0.09	*ρ* = 0.22	*ρ* = −0.12	*ρ* = −0.15	*ρ* = 0.03	*ρ* = 0.15

ρ, Spearman’s rank correlation coefficient. **p < 0.001, *p < 0.05.

### 3.4 Convergent validity: teacher ratings

The initial validity checks showed moderate correlations between teacher-rated and self-perceived prosocial behavior (*ρ* = 0.36). The correlation of teacher-rated emotion regulation skills was moderate for student-rated *ERS* for anxiety (*ρ* = 0.33), but low for anger (*ρ* = 0.20) and for sadness (*ρ* = 0.12).

The correlations between teacher ratings and student scores in *Internalizing Behavior* (*ρ* = 0.11) and *Externalizing Behavior* (*ρ* = 0.19) were low. Interestingly, there was a significant medium negative correlation between teacher-rated prosocial behavior and scores in externalizing behavior (*ρ* = −0.30) as well as teacher-rated prosocial behavior and *ERS* for anxiety (*ρ* = 0.38). Table 5 depicts all correlations.

**Table 5:** Correlations between teacher rating and student scores.

	Internalizing behavior^a^	Externalizing behavior^a^	Prosocial behavior^a^	Social withdrawal^a^	Emotion regulation^a^
GraSEF’s subtests^b^					
Internalizing behavior	*ρ* = 0.11	*ρ* = −0.07	*ρ* = −0.00	*ρ* = −0.07	*ρ* = 0.07
Externalizing behavior	*ρ* = 0.24^*^	*ρ* = 0.19	*ρ* = −0.30^*^	*ρ* = 0.04	*ρ* = −0.18
Problem-solving/assertive behavior	*ρ* = −0.07	*ρ* = −0.04	*ρ* = 0.22	*ρ* = −0.10	*ρ* = 0.09
Social withdrawal	*ρ* = 0.16	*ρ* = 0.24^*^	*ρ* = −0.11	*ρ* = −0.04	*ρ* = −0.12
Prosocial behavior^b^	*ρ* = −0.22	*ρ* = −0.09	*ρ* = 0.36^*^	*ρ* = −0.10	*ρ* = 0.28^*^
ERS: anger	*ρ* = −0.27^*^	*ρ* = 0.02	*ρ* = 0.23	*ρ* = 0.04	*ρ* = 0.20
ERS: sadness	*ρ* = −0.20	*ρ* = −0.04	*ρ* = 0.22	*ρ* = 0.09	*ρ* = 0.12
ERS: anxiety	*ρ* = −0.10	*ρ* = −0.28^*^	*ρ* = 0.38^**^	*ρ* = 0.20	*ρ* = 0.33^*^

^a^Teacher rating; ^b^student self-rating/scores; ERS, emotion regulation strategies. **p < 0.001, *p < 0.05.

### 3.5 Usability and user-friendliness

The completion of GraSEF took 28.74 min on average (*SD* = 6.28), varying from a minimum of 17.20 min to a maximum of 45.68 min. The mean time for each subtest ranged from *M* = 46.72 s (*SD* = 18.06, *Self-Perception of Emotions*) to *M* = 799.40 s (*SD* = 186.88, *Behavior in Social Situations*). The student feedback on the questions was positive. Out of the 68 participants, 56 children (82.4%) stated that they liked the questions. Two participants (2.9%) did not like them, while four (5.9%) rated question likability as average. Six children (8.8%) said that they liked the questions somewhat. The children most liked matching images to emotions (*Emotion Recognition*) and they particularly liked the pictures generated.

Through observation and brief interviews, we identified some necessary changes for further implementing the subtest *Behavior in Social Situations*. For Situation 8, we changed the original name Ana as a student had the same name and was irritated by it. For Situation 12, the protagonists were three friends and the feedback was that the answers would be different if they were “only” classmates. So, we changed the protagonists to ‘other children’ instead of friends, to get a more objective answer. One situation (Situation 5) was excluded as it was described as difficult to understand, and also exhibited insufficient distribution.

### 3.6 Exploratory analysis of gender differences

As an exploratory analysis, we investigated gender differences in the subtest *Behavior in Social Situations*. We conducted a MANOVA, which revealed a significant a main effect of gender [*F*(4, 63) = 8.22, *p* < 0.001, *η^2^* = 0.343]. To further examine the influence of gender on each subtest, we performed separate ANOVAs for each subscale, applying a Bonferroni correction to account for multiple comparisons (new alpha level: *α* = 0.013). The difference in *Externalizing Behavior* was significant between boys and girls, with boys showing a higher mean (*M* = 16.69, *SD* = 12.07) than girls (*M* = 9.47, *SD* = 7.01). The results for all subscales in this subtest are shown in Table 6.

**Table 6:** Gender differences in the subtest behavior in social situations.

Subscale	*F*(df)	*p*	*η^2^*	*M*_girls_ (*SD*)	*M*_boys_ (*SD*)
Behavior in social situations					
Internalizing behavior	3.18 (1, 66)	0.079	0.046	19.81 (7.93)	14.94 (13.52)
Externalizing behavior	8.82 (1, 66)	0.004*	0.118	9.47 (7.01)	16.69 (12.07)
Problem-solving/assertive behavior	6.07 (1, 66)	0.016	0.084	41.13 (5.37)	36.72 (8.75)
Social withdrawal	0.71 (1, 66)	0.403	0.011	22.41 (7.55)	24.31 (10.59)

Possible maximum of each subscale = 48. *Significant difference after Bonferroni correction (α = 0.013).

## 4 Discussion

The present study analyzed the newly developed digital screening instrument for assessing social–emotional skills (GraSEF) and investigated the procedure’s psychometric properties, such as reliability, validity and usability by making use of a sample of 68 second graders. The initial instrument was constructed on the basis of the CASEL framework and consisted of five subtests (1) *Behavior in Social Situations,* (2) *Prosocial Behavior*, (3) *Emotion Regulation Strategies (ERS),* (4) *Emotion Recognition,* and *(5) Self-Perception of Emotions*. In total, the screening instrument consisted of 10 subscales with 112 items.

The initial item analyses showed acceptable to good internal consistency for most of the subscales. The discriminatory power and the difficulty of the items were also within an acceptable range. However, one subtest (*Self-Perception of Emotions*) displayed very low internal consistency. We thus excluded this subtest and also removed some other items exhibiting insufficient distribution and very low discriminatory power. In the subtest *Emotion Recognition*, we removed image7 due to low concordance of students’ responses indicating high item difficulty. This image showed an anxious boy, but it may not have been the best representation of anxiety and would have required more extensive piloting. For the SJT (*Behavior in Social Situations*), we decided to exclude three situations (SV2, SV5, and SV9) due to insufficient variation in the answers. For two situations we had to change the names of the protagonists and some items had to be modified to eliminate the possibility of providing socially desirable responses. The final subtest encompasses various situations including someone taking one’s belongings, social exclusion, damaging property, joining a peer group, being teased by others, social assertiveness and dealing with other people’s mistakes. This then resulted in a total of four subtests and 89 items.

For the subtest *Behavior in Social Situations*, only on one subscale (*Social Withdrawal*) responses were normally distributed. The responses on the subscale *Problem-Solving/Assertive Behavior* were skewed to the left and on the subscales *Internalizing Behavior* and *Externalizing Behavior* they were skewed to the right. It can be concluded, that the children seem to prefer responses from one side, the majority of children appear to prefer the same direction, while only a few children demonstrate a tendency towards more individual responses. One potential reason for this is the social desirability of the children’s answers. For example, Camerini and Schulz (2018) found that children aged 9–10 tend to over-report positive and desirable behaviors and under-report negative behaviors, such as externalizing behaviors. Social desirability might have influenced the response behavior in the subtest *Behavior in Social Situations* (especially the subscale *Problem-Solving/Assertive Behavior*) and *Prosocial Behavior.* Although the screening was administered digitally and the children were not directly influenced by test administrators, they might already know which behavioral responses are desirable in social situations. To mitigate this in future studies, we suggest assessing social desirability directly using validated scales tailored to children. This result might also influence the inferences drawn from individual GraSEF results. While this assessment can reveal what children know about social situations (Dodge et al., 1985), it does not provide insights into how children actually react in these situations. Children with high scores in *Problem-Solving/Assertive Behavior* may understand social situations well and know how to react in these situations to be socially effective.

For other subtests, such as *ERS*, the item means were again not normally distributed, and the answers showed greater variance than that found in other subscales. Therefore, we conclude that social desirability did not influence response behavior in this subtest. This is supported by Dadds et al. (1998), who found no evidence that self-reported anxiety scores correlated with social desirability in a younger age group.

As far as the factor structure was concerned, the subtest *Behavior in Social Situations,* with its four subscales, and the subtest *ERS,* with its three subscales, showed the theoretically expected structures. For the subtest *Behavior in Social Situations* two items showed non-significant factor loadings for *Social Withdrawal* (SV6_su, SV13_su). A possible explanation for that is that these two items describe relatively active behaviors (picking up the pencil and giving in) rather than passive behaviors typically associated with social withdrawal.

The factor analysis for *ERS* showed a better fit for the three-factor model, but we found a high correlation between different *ERS*. This means that the different emotion regulation strategies are strongly related to each other – as already mentioned by Saarni et al. (2006). Thus, in future, shortening the screening instrument by concentrating on only one emotional dimension, such as anxiety, is well worth considering. For the other subtests and subscales, we only checked for, and confirmed, one-dimensionality. The factor analyses were difficult to interpret due to the small sample size and definite conclusions about the factorial structure cannot be made.

The validity check showed moderate to low correlations between the student and teacher ratings, depending on the different skills and behaviors. The correlations found in this study are consistent with those reported in other studies, that have investigated cross-informant agreement (e.g., Gresham et al., 2010; Gresham et al., 2018). This is also in line with Mudarra et al. (2022), who reported moderate correlations between teacher and student ratings of prosocial behavior. As students’ prosocial behavior is very important for positive social interactions in class, teachers are likely to be quite aware of students’ positive skills (Eisenberg et al., 2015). Moderate positive correlations were also found between teacher-rated emotion regulation and the subtest *ERS* in relation to students’ anxiety. This dimension may be more visible to teachers as they have many opportunities in the classroom to observe anxiety experienced by the students in specific situations. However, low correlations were observed for teacher and student ratings of emotion regulation for sadness and anger. The regulation of these emotions may be more difficult for teachers to observe. For example, Neil and Smith (2017) highlighted, that the correlations between teacher and student ratings for anxiety are often small, suggesting limited sensitivity of teachers to internalized emotions. Interestingly, students’ responses in the subtest *ERS* for anxiety correlated positively with teacher’s general rating of their emotion regulation. This may indicate that teachers might not directly detect anxiety, but their perception of a student’s emotion regulation skills are consistent with the specific responses obtained from the subtest. Also, noteworthy are the moderate correlations between teacher ratings of prosocial behavior and student ratings of *ERS* for anxiety. Prosocial behavior is likely to be associated with more positive ERS. For example, children who make greater use of ERS to cope with anxiety are more likely to be outgoing and to help others (Memmott-Elison et al., 2020).

We also found a negative correlation between teacher-rated externalizing behavior and student ratings of problem-solving/assertive behavior. This finding corresponds to the observations made by Huber et al. (2019). The higher the teachers rated their students’ externalizing behavior, the lower the students’ scores in problem-solving/assertive behavior in the subtest *Behavior in Social Situations*. Our study also showed a similar result for the self-reported problem-solving/assertive behavior and externalizing behavior. This highlights the link between problem behavior and other social–emotional skills (Bornstein et al., 2010). The correlation between internalizing and externalizing behavior was moderate, and was consistent with findings from other studies (Huber et al., 2019; Neil and Smith, 2017). The fact that other studies have come to similar findings, confirms the validity of the scales used here. It also supports the hypothesis that these dimensions of social–emotional skills can also be measured appropriately with adequate instruments in younger students.

As our instrument was the first German SEL instrument to be administered via tablets and audio files to young primary school students in a classroom setting, we also investigated its user-friendliness. SELweb (McKown, 2019) also is digital assessment tool using headphones and a mouse. However, there is no information available about its usability, except for the reported testing time. At 45 min, it took the children slightly longer than clicking trough GraSEF. The administration of GraSEF took about half an hour, although there was considerable variation here due to different student processing speeds. However, it should be possible to shorten the measurement procedure so that students are able to complete all items in about half an hour. Overall, the student feedback on the usability of the test was very positive. They understood the tasks and items, and they liked the item formats, especially the matching of pictures to statements (subtest *Emotion Recognition*). The students were guided through the GraSEF by audios. We observed that, irrespective of their individual reading ability, all students were able to participate successfully.

These observations support the assumption that using a digital approach (using tablets and headphones) to assess social–emotional skills is appropriate for students of this age, and that it is particularly suitable for students with low reading skills. We found that children are quite enthusiastic about using digital tools for such tasks, a finding which is in line with those of Blumenthal and Blumenthal (2020).

### 4.1 Limitations

Since we only evaluated the instrument using a small sample, the results need to be interpreted with caution and cannot be generalized. However, this sample aligns with guidelines for psychometric piloting research and should allow to identify preliminary trends for further validation (Johanson and Brooks, 2009). These trends provide a foundation for future studies, but more complex models are required and will be carried out during standardization. The factor analyses should be repeated using a larger sample (*n* = 500) in order to obtain comprehensive results regarding the proposed structure of the instrument. Owing to this limitation, we only used very simple models and did not analyze the factorial structure of the whole instrument.

In addition, although we observed the children throughout the procedure and were available to answer their questions, we cannot rule out the possibility that there were language or comprehension problems. To take this into account, we decided to exclude some children whose German was judged by the teacher to be rather low. Given the linguistic diversity in Austrian schools, the application of GraSEF to children with a first language other than German is an important consideration. Further research should investigate language adaptations to ensure wider use of GraSEF. There might also be cultural biases, which should be considered.

As the present research was a preliminary study designed to analyze the psychometric criteria of the scales, we did not use a standardized measurement for validation. We intend to do this in a subsequent step after the evaluation of the various scales and the final composition of the instrument has been completed. An analysis of instrumental fairness is also intended at some future point, i.e., an analysis entailing an examination of variances relating to language, gender and other socio-demographic variables.

Another limitation of the study is the reliance on teacher ratings for validation (Gresham et al., 2010; Gresham et al., 2018; Sointu et al., 2012). It has been shown, that correlations between teacher and student ratings tend to be low (Huber et al., 2019; Mudarra et al., 2022), and teacher ratings might be influenced by biases. In addition, teachers might have limited sensitivity in recognizing internalizing symptoms (Neil and Smith, 2017). To address this limitation in future studies, alternative or additional measures should be used. These could include more objective assessments, such as direct observations, or parent reports to provide a broader perspective on the social–emotional skills of the students.

## 5 Conclusion

The present study introduced the GraSEF (Screening to assess social–emotional skills), a digital assessment instrument in German, suitable for use with children in the early school years. It is based on the CASEL model and, after this initial validation, it consists of four subtests ((1) *Behavior in Social Situations,* (2) *Prosocial Behavior*, (3) *Emotion Regulation Strategies,* (4) *Emotion Recognition*) and 89 items. The GraSEF appears to be a promising instrument for providing practitioners with detailed insights into student social–emotional skills. It can be used to identify children at risk of behavior problems, particularly because of the under-recognition of internalizing problems by teachers (Neil and Smith, 2017). The use of an SJT to measure children’s social–emotional skills is a relatively new but promising approach, as demonstrated by Murano et al. (2021) and in this study. The SJT used in GraSEF is a direct assessment, which has an important role in assessing social–emotional competence because children demonstrate their skills in a specific task (McKown, 2019). Especially the digital administration of the instrument eases the implementation in inclusive classrooms. The gender differences found suggest that boys and girls show different response behaviors at this age, which should be taken into account in future measurement approaches, in the further development of this instrument, and in teacher training.

Further research includes administering GraSEF to a more diverse sample, exploring cross-cultural differences, testing it with younger and older children (e.g., first and third grade) and focusing on other psychometric analyses such as re-test reliability or divergent validity.

As a next immediate step, the instrument is to be analyzed in depth and standardized by making use of a larger sample size.

## Data Availability

The datasets presented in this study can be found in online repositories. The names of the repository/repositories and accession number(s) can be found below: Open Science Framework (OSF): https://osf.io/xd83n.

## Footnotes

^1^The “a.o.-status” (extraordinary status) is for students who have recently arrived in Austria and have a very low proficiency in German. They can have this status for a maximum of 2 years and do not receive grades throughout this time.
