# Supplementary material for: Digitally assessing social–emotional skills in early school years: initial validation of a screening instrument
Source: Front Psychol. 2025 Feb 6;16:1529083. doi: 10.3389/fpsyg.2025.1529083 (PMC11844663; doi:10.3389/fpsyg.2025.1529083)
Supplement: Supplementary file 1 [file Data_Sheet_1.PDF]

Supplementary Material A

Supplementary Tables (A): All items of the GraSEF

Table A1. Situations of Subtest 1: *Behavior in Social Situations*

	Situation (German, original)	Situation (English, translation)
SV1	In der Pause gehen alle Kinder in den Schulhof. Du freust dich schon und holst dir deinen Lieblingsball. Es liegen noch viele andere Bälle herum, aber Tom nimmt dir deinen Lieblingsball direkt vor der Nase weg. Was tust du?	At break time, all the children go out into the school playground. You’ve been looking forward to it and pick up your favorite ball. There are lots of other balls lying around, but Tom takes your favorite ball right out of your hand. What do you do?
SV2*	Du sitzt in der Klasse und suchst nach deinem Radiergummi. Auf einmal siehst du, dass Rob ihn auf seinem Tisch hat. Was tust du?	You're sitting in class looking for your eraser. Suddenly you see that Rob has it on his desk. What do you do?
SV3	Ihr macht einen Ausflug mit dem Bus. Du möchtest neben deinem besten Freund sitzen, denn die Busfahrt dauert eine Stunde. Jona, ein Mitschüler, hat sich aber bereits dort hingesetzt. Was tust du?	You are going on a bus trip. You want to sit next to your best friend because the bus ride takes an hour. But Jona, a classmate, has already taken the seat. What do you do?

SV4	Auf dem Schulhof ist viel los! Viele Kinder spielen in Gruppen. Du fragst Momo, ob du bei ihm mitspielen darfst. Er sagt: „Nein!“.Was tust du?	There's a lot going on in the school playground! Lots of children are playing in groups. You ask Momo if you can play with him. He says: ‘No!’. What do you do?
SV5*	Ami malt in dein Schulheft. Du siehst es und sagst: „Das darfst du nicht tun, das ist mein Heft!". Sie hört nicht auf und malt die ganze Heftseite voll. Was tust du?	Ami is drawing in your exercise book. You see this and say: ‘You shouldn’t do that, that's my exercise book!’. She doesn't stop and draws all over the page. What do you do?
SV6	Du hast einen neuen Buntstift. Er gefällt dir richtig gut. Lia kommt zu dir und möchte ihn ausborgen. Sie läuft damit herum und auf einmal fällt der Buntstift auf den Boden und die Spitze bricht ab. Was tust du?	You have a new colored pencil. You really like it. Lia comes to you and wants to borrow it. She walks around with it. Suddenly, the pencil falls to the floor and the tip breaks off. What do you do?
SV7	Du bist in deiner Klasse. Die Lehrerin bittet euch, euch für eine Gruppenarbeit in Gruppen einzuteilen. In der Klasse bilden sich Gruppen und niemand fragt dich, ob du mitmachen willst. Was tust du?	You are in your class. The teacher asks you to divide into groups for group work. Groups form in the class and nobody asks you if you want to take part. What do you do?
SV8[a]	Ana und Elif spielen gemeinsam im Schulhof. Du siehst die beiden lachen und möchtest auch mitspielen. Sie sehen dich an, fragen aber nicht, ob du mitspielen willst. Was tust du?	Ana and Elif are playing together in the school playground. You see them laughing and want to join in. They look at you but don't ask if you want to play. What do you do?
SV9*	Du möchtest in der Pause deine Jause essen. Manni ärgert dich ständig. Was tust du?	You want to eat your snack during the break. Manni keeps teasing you. What do you do?

SV10	Es ist Pause. Enes kommt zu dir und ihr streitet. Dann zieht Enes dich an deinen Haaren. Was tust du?	It's break time. Enes comes to you and you have an argument. Then Enes pulls your hair. What do you do?
SV11	Du bist im Schulhof und läufst sehr schnell. Es ist rutschig und du rutschst aus. Mimi, ein Kind aus deiner Klasse, sieht das und lacht. Was tust du?	You are in the school playground and running very fast. It's slippery and you slip. Mimi, a child in your class, sees this and laughs. What do you do?
SV12[a]	Du spielst mit deinen Freunden und Freundinnen Abfangen in der Pause. Es ist langweilig, du möchtest etwas Anderes spielen. Die anderen aber noch weiter spielen. Was tust du?	You are playing catch with your friends during the break. It's boring, you want to play something else,but the others want to continue. What do you do?
SV13	Du machst mit Vani gemeinsam die Rechenaufgabe. Ihr seid euch uneinig, welche Übung ihr zuerst machen wollt. Du möchtest mit der ersten und sie mit der letzten Übung beginnen. Was tust du?	You are doing the maths problem with Vani. You disagree about which exercise you should do first. You want to start with the first exercise and she wants to start with the last one. What do you do?
SV14	Du machst mit Sam gemeinsam ein Rätsel für die Schule. Beim Ausfüllen siehst du, dass Sam ein paar falsche Antworten eingetragen hat. Was tust du?	You and Sam are doing a puzzle together for school. When filling in the answers, you realize that Sam has a few answers wrong. What do you do?
SV15	Susi hält ein Referat über Bäume. Dir fällt auf, dass sie einen Fehler auf ihrem Plakat hat. Sie hat zwei Bäume verwechselt. Das stört dich sehr, weil die Kinder nun etwas Falsches lernen. Was tust du?	Susi is giving a presentation on trees. You notice that she has made a mistake on her poster. She has mixed up two trees. This really bothers you because the children are now learning the wrong thing. What do you do?

Supplementary Table A1. We used DeepL to translate the items. *Removed Situations. [a] Situations, that will be changed.

Table A2. Items of GraSEF: Subtest 1 - *Behavior in Social Situations: Internalizing Behavior*

Variable	Items (German, original)	Items (English, translation)
SV1_int	Ich bin traurig und gehe weg, weil ich damit spielen wollte.	I am sad and walk away because I wanted to play with it.
SV2_int*	Ich bin sehr traurig, weil Rob ihn einfach genommen hat.	I am very sad because Rob just took it.
SV3_int	Ich bin die ganze Busfahrt lang traurig.	I am sad throughout the whole bus ride.
SV4_int[a]	Ich bin sehr traurig und ziehe mich zurück.	I am very sad and withdraw.
SV5_int*	Ich bin traurig und beleidigt, weil mein Heft nun nicht mehr schön ist.	I am sad and offended because my notebook isn't pretty anymore.
SV6_int	Ich weine, weil Lia meinen Buntstift kaputt gemacht hat	I cry because Lia broke my pencil.
SV7_int[a]	Ich bin sehr traurig, weil ich mich ausgeschlossen fühle.	I am very sad because I feel excluded.
SV8_int	Ich bin sehr traurig, weil ich alleine bin.	I am very sad because I am alone.
SV9_int*	Es macht mich traurig, weil das total unfair ist.	It makes me sad because it's totally unfair.
SV10_int	Ich weine, weil es so weh tut.	I cry because it hurts so much.
SV11_int	Ich laufe schnell in die Schule und beginne zu weinen.	I quickly run to school and start crying.
SV12_int	Ich bin traurig und versuche, etwas anderes zu spielen.	I feel sad and try to play something else.
SV13_int	Ich bin traurig, weil wir nicht das gleiche wollen.	I am sad because we don't want the same thing.
SV14_int	Ich habe Angst, dass wir nun sehr wenig Punkte bekommen.	I am worried that we'll only get very few points now.
SV15_int	Ich habe Angst, etwas zu sagen.	I am afraid to say anything.

Supplementary Table A2. We used DeepL to translate the items. *Removed Items. [a] Items, that will be changed.

Table A3. Items of GraSEF: Subtest 1 - *Behavior in Social Situations: Externalizing Behavior*

Variable	Items (German, original)	Items (English, translation)
SV1_ext	Ich ärgere mich über ihn und schreie ihn an: „Gib mir den Ball sofort zurück!“	I get annoyed with him and shout at him: “Give me the ball back, immediately!”
SV2_ext*	Ich bin wütend und schreie Rob an, dass er mir den Radiergummi sofort zurückgeben soll.	I am angry and shout at Rob to immediately give me the eraser back.
SV3_ext[a]	Ich bin wütend und schreie Jona an: „Geh weg!“	I am angry and shout at Jona: “Go away!”
SV4_ext	Ich schreie ihn an: „Du bist gemein, ich lass dich auch nicht mehr mitspielen!“	I shout at him: “You're mean, I won't let you play anymore either!”
SV5_ext*	Ich schreie Ami an, schubse sie und nehme ihr mein Heft weg.	I shout at Ami, push her and take my notebook away from her.
SV6_ext	Ich bin wütend auf Lia und schreie sie an.	I am angry with Lia and shout at her.
SV7_ext	Ich ärgere mich und sage den anderen, dass sie gemein sind.	I get angry and tell the others they're being mean.
SV8_ext	Ich bin wütend und rufe: „Ihr seid gemein! Warum lasst ihr mich nicht mitspielen?“.	I am angry and shout: “You're mean! Why won't you let me play?”.
SV9_ext*	Ich haue Manni, damit er aufhört.	I hit Manni to make him stop.
SV10_ext	Ich schubse Enes, damit er aufhört.	I push Enes to make him stop.
SV11_ext	Ich bin richtig wütend auf Mimi und schubse sie.	I am really angry with Mimi and push her.
SV12_ext	Ich werde wütend und laut. Das ist so langweilig!	I get angry and loud. This is so boring!
SV13_ext	Ich werde laut und beginne einfach mit der ersten Übung.	I get loud and just start the first exercise.
SV14_ext[a]	Ich ärgere mich über Sam und schreie ihn an.	I get angry with Sam and shout at him.
SV15_ext	Ich rufe raus: „Susi, da ist ein Fehler!“.	I shout out, “Susi, there's a mistake!”.

Supplementary Table A3. We used DeepL to translate the items. *Removed Items. [a] Items, that will be changed.

Table A4. Items of GraSEF: Subtest 1 - *Behavior in Social Situations: Problem-Solving/Assertive Behavior*

Variable	Items (German, original)	Items (English, translation)
SV1_pro[a]	Ich frage, ob wir gemeinsam mit dem Ball spielen.	I ask if we can play with the ball together.
SV2_pro*	Ich frage Rob, ob er ihn mir zurückgeben kann.	I ask Rob if he can give it back to me.
SV3_pro[a]	Ich frage Jona, ob wir vielleicht tauschen können.	I ask Jona if we could swap seats.
SV4_pro[a]	Das macht nichts, ich frage eine Freundin, ob ich bei ihr mitspielen kann.	It doesn't matter, I ask a friend if I can play with her.
SV5_pro*	Ich erkläre Ami, dass das mein Heft ist und dass ich das nicht möchte.	I explain to Ami that this is my notebook and that I don't want her to do this.
SV6_pro	Ich sage zu Lia: „Bitte pass besser auf meine Sachen auf."	I tell Lia: "Please take better care of my things."
SV7_pro	Ich gehe zu einer Gruppe und frage, ob ich mitmachen kann.	I go to a group and ask if I can join them.
SV8_pro	Ich gehe zu den beiden hin und frage, ob ich mitspielen darf.	I approach them and ask if I can play with them.
SV9_pro*	Ich bitte Manni, mich in Ruhe zu lassen.	I ask Manni to leave me alone.
SV10_pro[a]	Ich bitte Enes, aufzuhören.	I ask Enes to stop.
SV11_pro[a]	Ich gehe zu Mimi hin und sage ihr: „Das ist nicht ok! Hör bitte auf!".	I go to Mimi and tell her: "That's not okay! Please stop!".
SV12_pro	Ich frage noch einmal, ob wir etwas anderes spielen können.	I ask again if we can play something else.
SV13_pro	Ich überlege mit Vani, wie wir zu einer Lösung kommen können.	I think about how Vani and I can find a solution.
SV14_pro[a]	Ich spreche mit ihm und schreibe die richtigen Lösungen hin.	I talk to him and write down the correct solutions.
SV15_pro	Ich sage ihr nach dem Referat, dass sie zwei Bäume verwechselt hat.	After the presentation, I tell her that she has mixed up two trees.

Supplementary Table A4. We used DeepL to translate the items. *Removed items. [a] Items, that will be changed.

Table A5. Items of GraSEF: Subtest 1 (Behavior in Social Situations: *Social Withdrawal*)

Variable	Items (German, original)	Items (English, translation)
SV1_su	Ich warte, bis Tom fertig gespielt hat und spiele später damit.	I wait until Tom has finished playing and play with it later.
SV2_su*	Ich warte, bis Rob von selbst zu mir kommt und mir den Radiergummi zurück gibt.	I wait until Rob comes to me by himself and gives me the eraser back.
SV3_su	Ich gehe weg und weiß nicht, was ich tun soll.	I leave and don't know what to do.
SV4_su	Ich setze mich einmal alleine auf die Bank.	I sit down on the bench on my own.
SV5_su*	Ich bin unsicher, was ich tun soll und lasse sie weitermachen.	I am unsure what to do and let them get on with it.
SV6_su	Ich hebe den Buntstift auf und sage nichts.	I pick up the pencil and say nothing.
SV7_su	Ich bleibe still sitzen und warte, ob mich noch jemand fragt.	I quietly remain seated and wait to see if anyone else asks me.
SV8_su[a]	Ich sage nichts und spiele einfach alleine.	I don't say anything and just play on my own.
SV9_su*	Ich gehe mit meiner Jause schnell wo anders hin.	I quickly go somewhere else with my snack.
SV10_su	Ich ignoriere Enes und laufe weg.	I ignore Enes and run away.
SV11_su	Ich gehe schnell von Mimi weg.	I quickly walk away from Mimi.
SV12_su	Ich spiele weiter, obwohl es für mich langweilig ist.	I keep playing even though it's boring for me.
SV13_su[a]	Ich gebe nach und lasse Vani entscheiden.	I give in and let Vani decide.
SV14_su	Ich sage gar nichts, obwohl ich die richtigen Antworten weiß.	I don't say anything, even though I know the right answers.
SV15_su	Ich sage nichts, obwohl mich der Fehler sehr stört.	I don't say anything, even though the mistake bothers me a lot.

Supplementary Table A5. We used DeepL to translate the items. *Removed items. [a] Items, that will be changed.

Table A6. Items of GraSEF: Subtest 2 (*Prosocial Behavior*)

Variable	Items (German, original)	Items (English, translation)
PV1	Wie oft hast du andere Kinder getröstet, wenn sie traurig waren?	How often have you comforted other children when they were sad?
PV2	Wie oft hast du anderen Kindern etwas Nettes gesagt?	How often have you said something nice to other children?
PV3	Wie oft hast du anderen Kindern in der Klasse geholfen?	How often have you helped other children in class?
PV4	Wie oft hast du anderen Kindern eine Freude gemacht?	How often have you made other children happy?
PV5	Wie oft hast du anderen Kindern etwas geborgt, wenn sie etwas vergessen haben?	How often have you lent other children something when they forgot something?

Supplementary Table A6. We used DeepL to translate the items.

Table A7. Items of GraSEF: Subtest 3 (*Emotion Regulation Strategies: Anger*)

Variable	Items (German, original)	Items (English, translation)
W1	Damit ich weniger wütend bin, mache ich etwas Lustiges.	To make myself less angry, I do something I enjoy.
W2	Damit ich weniger wütend bin, denke ich über Dinge nach, die mich fröhlich machen.	To make myself less angry, I think about things that make me happy.
W3	Damit ich weniger wütend bin, denke ich an schöne Dinge.	To make myself less angry, I think about nice things.
W4	Damit ich weniger wütend bin, denke ich an etwas anderes.	To make myself less angry, I think about something else.
W5	Damit ich weniger wütend bin, denke ich, dass die Wut schnell vorbei geht.	To make myself less angry, I think that the anger will quickly pass.
W6	Damit ich weniger wütend bin, sage ich mir, dass es nicht so schlimm ist.	To make myself less angry, I tell myself that it's not that bad.
W7	Damit ich weniger wütend bin, versuche ich, das Beste daraus zu machen.	To make myself less angry, I try to make the best of it.
W8	Damit ich weniger wütend bin, versuche ich das zu ändern, was mich wütend macht.	To make myself less angry, I try to change the thing that makes me angry.
W9	Damit ich weniger wütend bin, überlege ich, was ich gegen meine Wut tun kann.	To make myself less angry, I think about what I can do about my anger.
W10*	Damit ich weniger wütend bin, weiß ich nicht, was ich tun kann. (inv)	I don't know what to do to make myself less angry. (inv)
W11*	Damit ich weniger wütend bin, kann ich nichts tun. (inv)	There’s nothing I can do to make myself less angry. (inv)

Supplementary Table A7. We used DeepL to translate the items. *Removed items. Inv. = Inverted items.

Table A8. Items of GraSEF: Subtest 3 (*Emotion Regulation Strategies: Sadness*)

Variable	Items (German, original)	Items (English, translation)
T1	Damit ich weniger traurig bin, mache ich etwas Lustiges.	To make myself less sad, I do something I enjoy.
T2	Damit ich weniger traurig bin, denke ich über Dinge nach, die mich fröhlich machen.	To make myself less sad, I think about things that make me happy.
T3	Damit ich weniger traurig bin, denke ich an schöne Dinge.	To make myself less sad, I think about nice things.
T4	Damit ich weniger traurig bin, denke ich an etwas anderes.	To make myself less sad, I think about something else.
T5	Damit ich weniger traurig bin, denke ich, dass die Traurigkeit schnell vorbei geht.	To make myself less sad, I think that the sadness will pass quickly.
T6	Damit ich weniger traurig bin, sage ich mir, dass es nicht so schlimm ist.	To make myself less sad, I tell myself that it's not that bad.
T7	Damit ich weniger traurig bin, versuche ich, das Beste daraus zu machen.	To make myself less sad, I try to make the best of it.
T8	Damit ich weniger traurig bin, versuche ich das zu ändern, was mich traurig macht.	To make myself less sad, I try to change the thing that makes me sad.
T9	Damit ich weniger traurig bin, überlege ich, was ich gegen meine Traurigkeit tun kann.	To make myself less sad, I think about what I can do about my sadness.
T10*	Damit ich weniger traurig bin, weiß ich nicht, was ich tun kann. (inv)	I don’t know what I can do to make myself less sad. (inv)
T11*	Damit ich weniger traurig bin, kann ich nichts tun. (inv)	There’s nothing I can do to make myself less sad.(inv)

Supplementary Table A8. We used DeepL to translate the items. *Removed items. Inv. = Inverted items.

Table A9. Items of GraSEF: Subtest 3 (*Emotion Regulation Strategies: Anxiety*)

Variable	Items (German, original)	Items (English, translation)
A1	Damit ich weniger Angst habe, mache ich etwas Lustiges.	To make myself less afraid, I do something fun.
A2	Damit ich weniger Angst habe, denke ich über Dinge nach, die mich fröhlich machen.	To make myself less afraid, I think about things that make me happy.
A3	Damit ich weniger Angst habe, denke ich an schöne Dinge.	To make myself less afraid, I think about nice things.
A4	Damit ich weniger Angst habe, denke ich an etwas anderes.	To make myself less afraid, I think about something else.
A5	Damit ich weniger Angst habe, denke ich, dass die Angst schnell vorbei geht.	To make myself less afraid, I tell myself that the fear will pass quickly.
A6	Damit ich weniger Angst habe, sage ich mir, dass es nicht so schlimm ist.	To make myself less afraid, I tell myself that it's not that bad.
A7	Damit ich weniger Angst habe, versuche ich, das Beste daraus zu machen.	To make myself less afraid, I try to make the best of it.
A8	Damit ich weniger Angst habe, versuche ich das zu ändern, was mir Angst macht.	To make myself less afraid, I try to change the thing that makes me afraid.
A9	Damit ich weniger Angst habe, überlege ich, was ich gegen meine Angst tun kann.	To make myself less afraid, I think about what I can do about my fear.
A10*	Damit ich weniger Angst habe, weiß ich nicht, was ich tun kann. (inv)	I don't know what I can do to make myself less afraid. (inv)
A11*	Damit ich weniger Angst habe, kann ich nichts tun. (inv)	There's nothing I can do to make myself less afraid. (inv)

Supplementary Table A9. We used DeepL to translate the items. *Removed items. Inv. = Inverted items.

Supplementary Table A10. Items of GraSEF: Subtest 4 (*Emotion Recognition*)

Variable	Items (German, original)	Items (English, translation)
Image1	Bild: Junge, fröhlich	Image: boy, happy
Image2	Bild: Mädchen, fröhlich	Image: girl, happy
Image3	Bild: Junge, traurig	Image: boy, sad
Image4	Bild: Mädchen, traurig	Image: girl, sad
Image5	Bild: Junge, wütend	Image: boy, angry
Image6	Bild: Mädchen, wütend	Image: girl, angry
Image7*	Bild: Junge, ängstlich	Image: boy, anxious
Image8	Bild: Mädchen, ängstlich	Image: girl, anxious
Image9	Bild: Junge, überrascht	Image: girl, surprised
Image10	Bild: Mädchen, überrascht	Image: boy, surprised

Supplementary Table A10. *Removed image.

Table A11. Items of GraSEF: Subtest 5 (*Self-Perception of Emotions*)

Variable	Items (German, original)	Items (English, translation)
E1*	Wie fühlst du dich, wenn dir dein Spielzeug von einem anderen Kind weggenommen wird? (Antwortmöglichkeiten: fröhlich, wütend, ängstlich)	How do you feel when your toy is taken away by another child? (possible answers: happy, angry, anxious)
E2*	Wie fühlst du dich, wenn du ein tolles Geschenk bekommst? (Antwortmöglichkeiten: fröhlich, traurig, wütend)	How do you feel when you get a great present? (possible answers: happy, sad, angry)
E3*	Wie fühlst du dich, wenn dein neuer Lieblingsbleistift kaputt wird? (Antwortmöglichkeiten: traurig, fröhlich, ängstlich)	How do you feel when your new favorite pencil breaks? (possible answers: sad, happy, anxious)
E4*	Wie fühlst du dich, wenn niemand mit dir spielen möchte? (Antwortmöglichkeiten: fröhlich, traurig, ängstlich)	How do you feel when no one wants to play with you? (possible answers: happy, sad, anxious)

Supplementary Table A11. We used DeepL to translate the items. *Removed items. Inv. = Inverted items.
