# Supplementary material for: Digitally assessing social–emotional skills in early school years: initial validation of a screening instrument
Source: Front Psychol. 2025 Feb 6;16:1529083. doi: 10.3389/fpsyg.2025.1529083 (PMC11844663; doi:10.3389/fpsyg.2025.1529083)
Supplement: Supplementary file 2 [file Data_Sheet_2.PDF]

Supplementary Material B

Supplementary Tables (B): Item analyses

Table B1. Item Analysis of the Subscale *Internalizing Behavior*

Item	*M*	*SD*	skew	kurtosis	SE	σ^2	P_i	$r_{i(t-i)}$ [a]	$r_{i(t-i)}$ [b]
SV1_int	1.69	1.66	0.32	-1.55	0.20	2.75	.42	.53	.51
SV2_int*	1.76	1.70	0.18	-1.70	0.21	2.90	.44	.60	-
SV3_int	1.12	1.43	0.91	-0.62	0.17	2.05	.28	.65	.66
SV4_int	1.59	1.51	0.35	-1.31	0.18	2.28	.40	.60	.60
SV5_int*	2.24	1.66	-0.27	-1.59	0.20	2.75	.56	.62	-
SV6_int	1.35	1.52	0.63	-1.13	0.18	2.23	.34	.52	.51
SV7_int	1.82	1.55	0.15	-1.51	0.19	2.42	.46	.55	.55
SV8_int	1.62	1.53	0.35	-1.34	0.19	2.33	.40	.63	.59
SV9_int*	1.96	1.55	0.05	-1.46	0.19	2.40	.49	.71	-
SV10_int	1.66	1.58	0.29	-1.48	0.19	2.50	.42	.55	.53
SV11_int	0.93	1.41	1.11	-0.35	0.17	1.98	.23	.55	.56
SV12_int	1.56	1.58	0.42	-1.37	0.19	2.49	.39	.54	.51
SV13_int	1.26	1.47	0.76	-0.87	0.18	2.17	.32	.62	.61
SV14_int	1.49	1.61	0.52	-1.37	0.19	2.58	.37	.38	.35
SV15_int	1.15	1.44	0.87	-0.71	0.17	2.07	.29	.46	.42

Supplementary Table B1. P_i = item difficulty, $r_{i(t-i)}$ = discriminatory power, *removed items [a] discriminatory power of item with all items, [b]discriminatory power of item after removing SV2_int, SV5_int, SV9_int

Table B2. Item Analysis of the Subscale *Externalizing Behavior*

Item	*M*	*SD*	skew	kurtosis	SE	σ^2	P_i	$r_{i(t-i)}$ [a]	$r_{i(t-i)}$ [b]
SV1_ext	0.91	1.42	1.20	-0.08	0.17	2.02	.23	.48	.47
SV2_ext*	0.87	1.43	1.43	0.46	0.17	2.06	.22	.50	-
SV3_ext	0.50	1.15	2.25	3.74	0.14	1.33	.12	.57	.57
SV4_ext	1.19	1.58	0.93	-0.82	0.19	2.49	.30	.61	.58
SV5_ext*	1.07	1.46	1.01	-0.50	0.18	2.13	.27	.53	-
SV6_ext	1.12	1.50	1.00	-0.55	0.18	2.25	.28	.56	.55
SV7_ext	0.87	1.36	1.40	0.56	0.16	1.85	.22	.60	.60
SV8_ext	0.96	1.44	1.14	-0.25	0.17	2.07	.24	.36	.40
SV9_ext*	0.93	1.51	1.25	-0.16	0.18	2.28	.23	.51	-
SV10_ext	1.53	1.67	0.49	-1.45	0.20	2.79	.38	.65	.60
SV11_ext	1.38	1.64	0.65	-1.27	0.20	2.69	.35	.60	.57
SV12_ext	0.79	1.30	1.54	1.09	0.16	1.69	.20	.51	.51
SV13_ext	1.53	1.71	0.55	-1.47	0.21	2.91	.38	.39	.38
SV14_ext	0.63	1.30	1.85	1.86	0.16	1.70	.16	.43	.42
SV15_ext	1.88	1.77	0.11	-1.77	0.21	3.12	.47	.27	.27

Supplementary Table B2. P_i = item difficulty, $r_{i(t-i)}$ = discriminatory power, *removed items [a]discriminatory power of item with all items, [b]discriminatory power of item after removing SV2_ext, SV5_ext, SV9_ext

Table B3. Item Analysis of the Subscale *Problem-Solving/Assertive Behavior*

Item	*M*	*SD*	skew	kurtosis	SE	σ^2	P_i	$r_{i(t-i)}$ [a]	$r_{i(t-i)}$ [b]
SV1_pro	3.32	1.18	-1.61	1.47	0.14	1.39	.83	.31	.26
SV2_pro*	3.54	1.04	-2.53	5.48	0.13	1.09	.89	.41	-
SV3_pro	3.16	1.42	-1.51	0.71	0.17	2.02	.79	.45	.46
SV4_pro	3.09	1.34	-1.20	0.07	0.16	1.78	.77	.39	.39
SV5_pro*	3.37	1.18	-2.00	2.92	0.14	1.40	.84	.48	-
SV6_pro	3.26	1.25	-1.62	1.41	0.15	1.57	.82	.38	.24
SV7_pro	3.32	1.25	-1.79	1.86	0.15	1.57	.83	.43	.39
SV8_pro	3.29	1.36	-1.62	0.99	0.17	1.85	.82	.42	.43
SV9_pro*	3.32	1.31	-1.74	1.55	0.16	1.71	.83	.58	-
SV10_pro	3.34	1.30	-1.80	1.75	0.16	1.69	.83	.23	.22
SV11_pro	3.38	1.11	-2.08	3.59	0.13	1.22	.85	.45	.44
SV12_pro	3.32	1.19	-1.63	1.45	0.14	1.42	.83	.48	.50
SV13_pro	2.99	1.40	-1.17	-0.01	0.17	1.96	.75	.33	.34
SV14_pro	3.18	1.32	-1.49	0.87	0.16	1.73	.79	.20	.17
SV15_pro	3.13	1.30	-1.40	0.72	0.16	1.70	.78	.37	.39

Supplementary Table B3. P_i = item difficulty, $r_{i(t-i)}$ = discriminatory power, *removed items [a]discriminatory power of item with all items, [b]discriminatory power of item after removing SV2_pro, SV5_pro, SV9_pro

Table B4. Item Analysis of the Subscale *Social Withdrawal*

Item	*M*	*SD*	skew	kurtosis	SE	σ^2	P_i	$r_{i(t-i)}$ [a]	$r_{i(t-i)}$ [b]
SV1_su	2.62	1.52	-0.61	-1.14	0.18	2.30	.65	.45	.41
SV2_su*	1.72	1.60	0.30	-1.47	0.19	2.56	.43	.37	-
SV3_su	1.38	1.52	0.69	-0.98	0.18	2.30	.35	.39	.38
SV4_su	2.15	1.51	-0.19	-1.39	0.18	2.28	.54	.40	.42
SV5_su*	0.54	1.21	2.09	2.96	0.15	1.48	.14	.43	-
SV6_su	1.41	1.43	0.63	-0.94	0.17	2.04	.35	.24	.17
SV7_su	2.01	1.65	-0.04	-1.62	0.20	2.73	.50	.62	.57
SV8_su	1.91	1.59	0.14	-1.55	0.19	2.53	.48	.45	.49
SV9_su*	2.56	1.41	-0.56	-0.92	0.17	1.98	.64	.16	-
SV10_su	2.09	1.75	-0.08	-1.76	0.21	3.07	.52	.32	.31
SV11_su	2.38	1.56	-0.43	-1.36	0.19	2.45	.60	.38	.35
SV12_su	1.91	1.57	0.14	-1.49	0.19	2.47	.48	.32	.31
SV13_su	2.69	1.35	-0.69	-0.68	0.16	1.83	.67	.19	.18
SV14_su	1.37	1.58	0.64	-1.20	0.19	2.50	.34	.35	.34
SV15_su	1.49	1.60	0.49	-1.40	0.19	2.55	.37	.27	.32

Supplementary Table B4. P_i = item difficulty, $r_{i(t-i)}$ = discriminatory power, *removed items [a]discriminatory power of item with all items, [b]discriminatory power of item after removing SV2_su, SV5_su, SV9_su

Table B5. Item Analysis of the Subscale *Prosocial Behavior*

Item	*M*	*SD*	skew	kurtosis	SE	σ^2	P_i	$r_{i(t-i)}$~~a~~
PV1	2.32	1.32	-0.25	-1.06	0.16	1.74	.58	.44
PV2	3.04	1.13	-1.14	0.48	0.14	1.27	.76	.40
PV3	3.09	0.99	-0.81	-0.08	0.12	0.98	.77	.52
PV4	2.71	1.07	-0.72	-0.03	0.13	1.14	.68	.60
PV5	2.88	1.10	-0.57	-0.80	0.13	1.21	.72	.40

Supplementary Table B5. P_i = item difficulty, $r_{i(t-i)}$ = discriminatory power

Table B6. Item Analysis of the Subscale *Emotion Regulation Strategies: Anger*

Item	*M*	*SD*	skew	kurtosis	SE	σ^2	P_i	$r_{i(t-i)}$ [a]	$r_{i(t-i)}$ [b]
W1	2.37	1.40	-0.34	-1.15	0.17	1.96	.59	.51	.55
W2	2.49	1.49	-0.53	-1.17	0.18	2.22	.62	.76	.78
W3	2.32	1.39	-0.42	-1.11	0.17	1.92	.58	.55	.57
W4	2.26	1.35	-0.23	-1.14	0.16	1.81	.57	.60	.59
W5	2.03	1.47	0.03	-1.36	0.18	2.14	.51	.71	.70
W6	1.94	1.40	0.01	-1.28	0.17	1.97	.49	.60	.62
W7	2.44	1.30	-0.48	-0.83	0.16	1.68	.61	.68	.64
W8	2.41	1.40	-0.55	-0.92	0.17	1.95	.60	.51	.52
W9	2.38	1.26	-0.38	-0.72	0.15	1.58	.60	.57	.55
W10 (inv)*	2.51	1.41	-0.38	-1.26	0.17	1.98	.63	-.06	-
W11 (inv)*	2.78	1.36	-0.76	-0.78	0.16	1.84	.70	.03	-

Supplementary Table B6. P_i = item difficulty, $r_{i(t-i)}$ = discriminatory power, inv = inverted item, *removed items, [a]discriminatory power of item with all items, [b]discriminatory power of item after removing W10, W11

Table B7. Item Analysis of the Subscale *Emotion Regulation Strategies: Sadness*

Item	*M*	*SD*	skew	kurtosis	SE	σ^2	P_i	$r_{i(t-i)}$ [a]	$r_{i(t-i)}$ [b]
T1	2.24	1.55	-0.25	-1.45	0.19	2.39	.56	.47	.47
T2	2.37	1.37	-0.26	-1.19	0.17	1.88	.59	.69	.62
T3	2.04	1.48	-0.16	-1.40	0.18	2.19	.51	.54	.56
T4	2.35	1.32	-0.35	-0.96	0.16	1.75	.59	.61	.63
T5	2.35	1.43	-0.23	-1.32	0.17	2.05	.59	.61	.63
T6	2.26	1.50	-0.24	-1.39	0.18	2.26	.57	.56	.62
T7	2.19	1.38	-0.21	-1.13	0.17	1.92	.55	.48	.55
T8	2.21	1.51	-0.24	-1.39	0.18	2.29	.55	.57	.64
T9	2.09	1.36	-0.16	-1.20	0.16	1.84	.52	.50	.60
T10 (inv)*	2.37	1.47	-0.33	-1.29	0.18	2.15	.59	.03	-
T11 (inv)*	2.43	1.40	-0.52	-0.98	0.17	1.95	.61	-.09	-

Supplementary Table B7. P_i = item difficulty, $r_{i(t-i)}$ = discriminatory power, inv = inverted item, *removed items, [a]discriminatory power of item with all items, [b]discriminatory power of item after removing T10, T11

Table B8. Item Analysis of the Subscale *Emotion Regulation Strategies: Anxiety*

Item	*M*	*SD*	skew	kurtosis	SE	σ^2	P_i	$r_{i(t-i)}$[a]	$r_{i(t-i)}$[b]
A1	2.60	1.41	-0.56	-1.03	0.17	1.97	.65	.54	.58
A2	2.51	1.24	-0.47	-0.72	0.15	1.54	.63	.72	.71
A3	2.46	1.45	-0.49	-1.14	0.18	2.10	.62	.65	.66
A4	2.25	1.39	-0.41	-1.08	0.17	1.92	.56	.58	.61
A5	1.93	1.51	0.02	-1.49	0.18	2.28	.48	.58	.63
A6	1.90	1.32	0.15	-1.07	0.16	1.74	.48	.52	.61
A7	2.21	1.31	-0.22	-0.99	0.16	1.72	.55	.56	.60
A8	2.29	1.41	-0.21	-1.25	0.17	2.00	.57	.61	.63
A9	2.18	1.21	-0.18	-0.59	0.15	1.46	.54	.55	.60
A10 (inv)*	2.19	1.49	-0.27	-1.36	0.18	2.22	.55	-.06	-
A11 (inv)*	2.38	1.50	-0.39	-1.25	0.18	2.24	.60	-.10	-

Supplementary Table B8. P_i = item difficulty, $r_{i(t-i)}$ = discriminatory power, inv = inverted item, *removed items, [a]discriminatory power of item with all items, [b]discriminatory power of item after removing A10, A11

Table B9. Item Analysis of the Subscale *Emotion Recognition*

Item	P_i
Image1 (boy, happy)	0.94
Image2 (girl, happy)	0.96
Image3 (boy, sad)	0.82
Image4 (girl, sad)	0.97
Image5 (boy, angry)	0.97
Image6 (girl, angry)	0.99
Image7 (boy, anxious)*	0.59
Image8 (girl, anxious)	0.78
Image9 (boy, surprised)	0.85
Image10 (girl, surprised)	0.75

Supplementary Table B9. P_i = item difficulty, *removed items
