# Supplementary material for: Digitally assessing social–emotional skills in early school years: initial validation of a screening instrument
Source: Front Psychol. 2025 Feb 6;16:1529083. doi: 10.3389/fpsyg.2025.1529083 (PMC11844663; doi:10.3389/fpsyg.2025.1529083)
Supplement: Supplementary file 3 [file Data_Sheet_3.PDF]

Supplementary Material C

Table C1. Correlations of all subscales.

	1	2	3	4	5	6	7	8	9
1 internal. behavior	-								
2 externalizing behavior	ρ = .46 (p < .001)	-							
3 problem-solving/ assertive behavior	ρ = .09 (p = .479)	ρ = - .40 (p < .001)	-						
4 social withdrawal	ρ = .49 (p < .001)	ρ = .33 (p = .007)	ρ = .19 (p = .131)	-					
5 prosocial behavior	ρ = -.05 (p = .698)	ρ = .04 (p = .757)	ρ = .16 (p = .181)	ρ = -.07 (p = .569)	-				
6 ERS: anger	ρ = .13 (p = .295)	ρ = .04 (p = .737)	ρ = .28 (p = .017)	ρ = .28 (p = .023)	ρ = .40 (p < .001)	-			
7 ERS: sadness	ρ = .16 (p = .189)	ρ = .09 (p = .485)	ρ = .23 (p = .056)	ρ = .18 (p = .138)	ρ = .33 (p = .006)	ρ = .80 (p < .001)	-		
8 ERS: anxiety	ρ = .11 (p = .363)	ρ = -.03 (p = .800)	ρ = .31 (p = .011)	ρ = .12 (p = .341)	ρ = .38 (p = .001)	ρ = .58 (p < .001)	ρ = .70 (p < .001)	-	
9 Emotion Recogntion	ρ = .10 (p = .416)	ρ = -.16 (p = .176)	ρ = .03 (p = .783)	ρ = -.04 (p = .775)	ρ = -.02 (p = .892)	ρ = .10 (p = .400)	ρ = .00 (p = .974)	ρ = -.03 (p = .780)	-
10 Emotion Perception	ρ = .20 (p = .101)	ρ = -.10 (p = .400)	ρ = .22 (p = .067)	ρ = -.09 (p = .456)	ρ = .22 (p = .078)	ρ = -.12 (p = .325)	ρ = -.15 (p = .211)	ρ = .03 (p = .808)	ρ = .15 (p = .222)

Supplementary Table C1. internal. = internalizing. ERS = emotion regulation strategies.

Table C2. Correlations of the subtests (b) with teacher ratings (a) and p-values

	internalizing behavior[a]	externalizing behavior[a]	prosocial behavior[a]	social withdrawal[a]	emotion regulation[a]
internalizing behavior[b]	ρ = .11 (p = .355)	ρ = -.07 (p = .561)	ρ = -.00 (p = .986)	ρ = -.07 (p = .568)	ρ = .07 (p = .590)
externalizing behavior[b]	ρ = .24 (p = .049)	ρ = .19 (p = .127)	ρ = -.30 (p = .014)	ρ = .04 (p = .754)	ρ = -.18 (p = .151)
problem-solving/assertive behavior[b]	ρ = -.07 (p = .558)	ρ = -.04 (p = .749)	ρ = .22 (p = .069)	ρ = -.10 (p = .417)	ρ = .09 (p = .467)
social withdrawal[b]	ρ = .16 (p = .197)	ρ = .24 (p = .050)	ρ = -.11 (p = .382)	ρ = -.04 (p = .753)	ρ = -.12 (p = .319)
prosocial behavior[b]	ρ = -.22 (p = .069)	ρ = -.09 (p = .461)	ρ = .36 (p = .003)	ρ = -.10 (p = .430)	ρ = .28 (p = .023)
ERS: anger[b]	ρ = -.27 (p = .026)	ρ = .02 (p = .901)	ρ = .23 (p = .054)	ρ = .04 (p = .745)	ρ = .20 (p = .100)
ERS: sadness [b]	ρ = -.20 (p = .103)	ρ = -.04 (p = .747)	ρ = .22 (p = .073)	ρ = .09 (p = .526)	ρ = .12 (p = .342)
ERS: anxiety [b]	ρ = -.10 (p = .426)	ρ = -.28 (p = .022)	ρ = .38 (p = .001)	ρ = .20 (p = .095)	ρ = .33 (p = .005)

Supplementary Table C2. [a] teacher-rating, [b] student self-rating. ERS = emotion regulation strategies.
